# An emerging simple and effective approach to increase the productivity of thraustochytrids microbial lipids by regulating glycolysis process and triacylglycerols’ decomposition

**DOI:** 10.1186/s13068-021-02097-4

**Published:** 2021-12-31

**Authors:** Wang Ma, Yu-Zhou Wang, Fang-Tong Nong, Fei Du, Ying-Shuang Xu, Peng-Wei Huang, Xiao-Man Sun

**Affiliations:** School of Food Science and Pharmaceutical Engineering, Nanjing Normal University, 2 Xuelin Road, Qixia District, Nanjing, People’s Republic of China

**Keywords:** Lipase inhibitor, *Schizochytrium* sp., Lipid productivity, Glucose-to-lipid conversion rater, Triacylglycerol

## Abstract

**Background:**

The oleaginous microorganism *Schizochytrium* sp. is widely used in scientific research and commercial lipid production processes. However, low glucose-to-lipid conversion rate (GLCR) and low lipid productivity of *Schizochytrium* sp. restrict the feasibility of its use.

**Results:**

Orlistat is a lipase inhibitor, which avoids triacylglycerols (TAGs) from hydrolysis by lipase. TAGs are the main storage forms of fatty acids in *Schizochytrium* sp. In this study, the usage of orlistat increased the GLCR by 21.88% in the middle stage of fermentation. Whereas the productivity of lipid increased 1.34 times reaching 0.73 g/L/h, the saturated fatty acid and polyunsaturated fatty acid yield increased from 21.2 and 39.1 to 34.9 and 48.5 g/L, respectively, indicating the advantages of using a lipase inhibitor in microbial lipids fermentation. Similarly, the system was also successful in Thraustochytrid *Aurantiochytrium*. The metabolic regulatory mechanisms stimulated by orlistat in *Schizochytrium* sp. were further investigated using transcriptomics and metabolomics. The results showed that orlistat redistributed carbon allocation and enhanced the energy supply when inhibiting the TAGs’ degradation pathway. Therefore, lipase in *Schizochytrium* sp. prefers to hydrolyze saturated fatty acid TAGs into the β-oxidation pathway.

**Conclusions:**

This study provides a simple and effective approach to improve lipid production, and makes us understand the mechanism of lipid accumulation and decomposition in *Schizochytrium* sp., offering new guidance for the exploitation of oleaginous microorganisms.

**Supplementary Information:**

The online version contains supplementary material available at 10.1186/s13068-021-02097-4.

## Background

In recent years, microbial lipids have received increased attention as an attractive renewable source for producing nutraceuticals, biodiesel, nutrition lipids, and functional foods [1]. For example, nutritional lipids, such as polyunsaturated fatty acids (PUFAs), which improve clinical outcomes of patients with acute respiratory distress syndrome and modulate the COVID-19 anti-inflammatory response [2]; biodiesel, which refers to saturated fatty acids (SFAs), are a class of renewable resource chemicals with high hopes of replacing fossil fuel-based products for greener development [3]. Microbial lipids yields from different types of oleaginous microbes range from 20 to 70% [4]. However, the economic feasibility of microbial lipids production is still uncertain due to high costs, so it is urgent to produce microbial lipids more economically and efficiently.

*Schizochytrium* sp., belonging to the Thraustochytriaceae family, can accumulate significant amounts of lipids [5]. However, high substrate costs and low lipid productivity are the biggest challenges facing the large-scale production of microbial lipids [6]. Traditionally, many studies have sought to improve the lipid production of the *thraustochytrids* through the optimization of fermentation processes. According to the related literature reports, lipid production by *Aurantiochytrium* sp. T66 was significantly enhanced when the cells were subjected to nitrogen limitation [7]. In addition, when the dissolved oxygen level was maintained at 50% throughout the whole fermentation process, *Aurantiochytrium limacinum* SR21 produced lipids at 5.75 g/L/day [8]. However, the technology has reached a point where lipid productivity cannot be further improved using fermentation optimization alone. Moreover, many studies have only focused on overall lipid production while ignoring the decrease in lipid productivity in the middle and late stages of fermentation, as well as the glucose-to-lipid conversion rate (GLCR). For example, a report investigated the impact that adjusting the pH with ammonia and citric acid can increase the lipid production of *Schizochytrium* sp. by 27.5%, but the lipid productivity and GLCR did not improve during the middle stage of fermentation (48–96 h), which may also be the cause of reaching a peak in production [9]. With the rise of synthetic biology, editing the genome of microorganisms to improve lipid production is also an increasingly viable strategy [10]. The expression of *Vitreoscilla stercoraria* hemoglobin in *Aurantiochytrium* sp. SK4 showed that the fatty acid content of the engineered strain was 44% higher than that of the wild type [11]. Furthermore, a recent study showed that the whole polyketide synthase (PKS) pathway from *Shewanella japonica* was expressed in *Aurantiochytrium*, resulting in a 26.9% increase in the lipid yield [12]. Up to date, nine genera of thraustochytrids have been recognized [13], however, efficient gene editing tools suitable for editing thraustochytrids have not been established. Therefore, the search for a simple and effective metabolic flow disturbance strategy has become the recent research priorities.

In recent years, more and more small-molecule drugs were found to disrupt the certain biological systems by targeting specific enzyme proteins or acting as signal molecules, thereby causing specific phenotypes of native producers [14]. These small-molecule drugs that act as activators or inhibitors are called chemical modulators. Many microorganisms that lack genetic engineering tools have benefited from chemical modulators in their lipid production [15]. The biosynthesis of microbial lipids relies on a powerful supply of adenosine triphosphate (ATP), acetyl-CoA, and nicotinamide adenine dinucleotide (NADPH), and the availability of substrates is the key to balance growth and lipid production as these two processes compete with each other [16]. Recently, most chemical modulators have been used to enhance the supply of acetyl-CoA and NADPH. For example, ethanolamine indirectly participates in the synthesis of fatty acids by regulating acetyl-CoA production. In *Scenedesmus obliquus*, ethanolamine can increase lipid production by 22%, which is considered a potential marker for improving lipid accumulation [17]. The supply of NADPH in the lipid synthesis process of eukaryotic microbial cells is mainly related to NADP-malic enzymes and enzymes in the pentose phosphate pathway. Liu et al. used inositol to strengthen the supply of NADPH by increasing NADP-malic enzyme activity, which resulted in a 13.9% increase in the lipid content of *Schizochytrium* sp. SR21 [18]. Nevertheless, most studies only focused on lipid accumulation, but the problem of lipid degradation cannot be ignored. Many studies have confirmed that the restriction of lipase function plays a significant role in promoting the accumulation of triacylglycerols (TAGs) in *Schizosaccharomyces pombe*, *Mucor circinelloides*, and *Thalassiosira pseudonana* [19–21].

Herein, five chemical modulators of metabolic pathways were selected and their effects were studied. Then, our study focused on the effect of lipase inhibitor orlistat on the lipid accumulation in *Schizochytrium* sp. HX-308. On this basis, we compared the antioxidant capacity of *Schizochytrium* sp. with or without orlistat by measuring reactive oxygen species (ROS), NADPH and total antioxidant capacity (T-AOC). Finally, the performances were systematically explained through transcriptomic and metabolomic analyses. The results of this study were expected to further our understanding of *Schizochytrium* sp. and provide new guidance for microbial lipid production.

## Results

### Screening chemical modulators and optimization of fermentation conditions

**Fig. 1:**
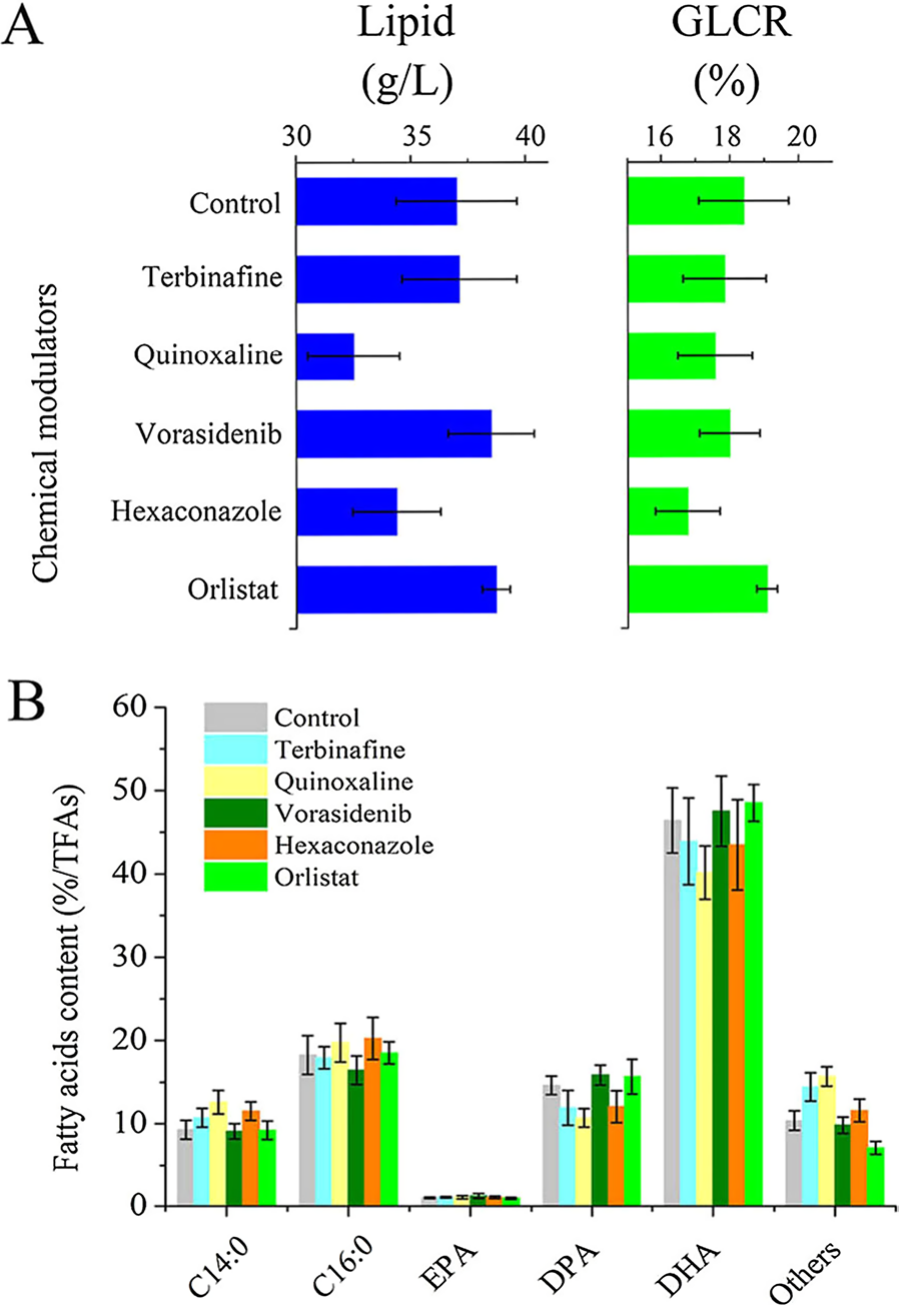
The influence of different chemical modulators on the lipid yield, GLCR (**A**) and fatty acids content (**B**) of *Schizochytrium* sp. HX-308

The effect of five selected chemical modulators on lipid accumulation in *Schizochytrium* sp. were studied (Additional file 1: Table S1). For the evaluation of their performance, lipid yield, GLCR and fatty acids content were used as indicators. After 3 days of fermentation, the addition of vorasidenib and terbinafine slightly promoted lipid accumulation (37.13 and 37.8 g/L vs 37.01 g/L), which is in line with our expectations, but the GLCR did not improve (Fig. 1). Interestingly, the addition of vorasidenib led to an increase in the proportion of PUFAs to 64.58%, in which the proportion of EPA was 1.24 times that of the control, but the addition of terbinafine had a limited effect on fatty acid content. In contrast, the addition of quinoxaline and hexaconazole negatively affected the lipid accumulation and GLCR, but the percentage of SFAs was increased by 17.32% and 15.27%, respectively. Unexpectedly, the lipase inhibitor orlistat produced different results: the lipid yield and GLCR were significantly higher than the control, and encouraged *Schizochytrium* sp. to accumulate more PUFAs (Additional file 1: Fig. S1).

**Fig. 2:**
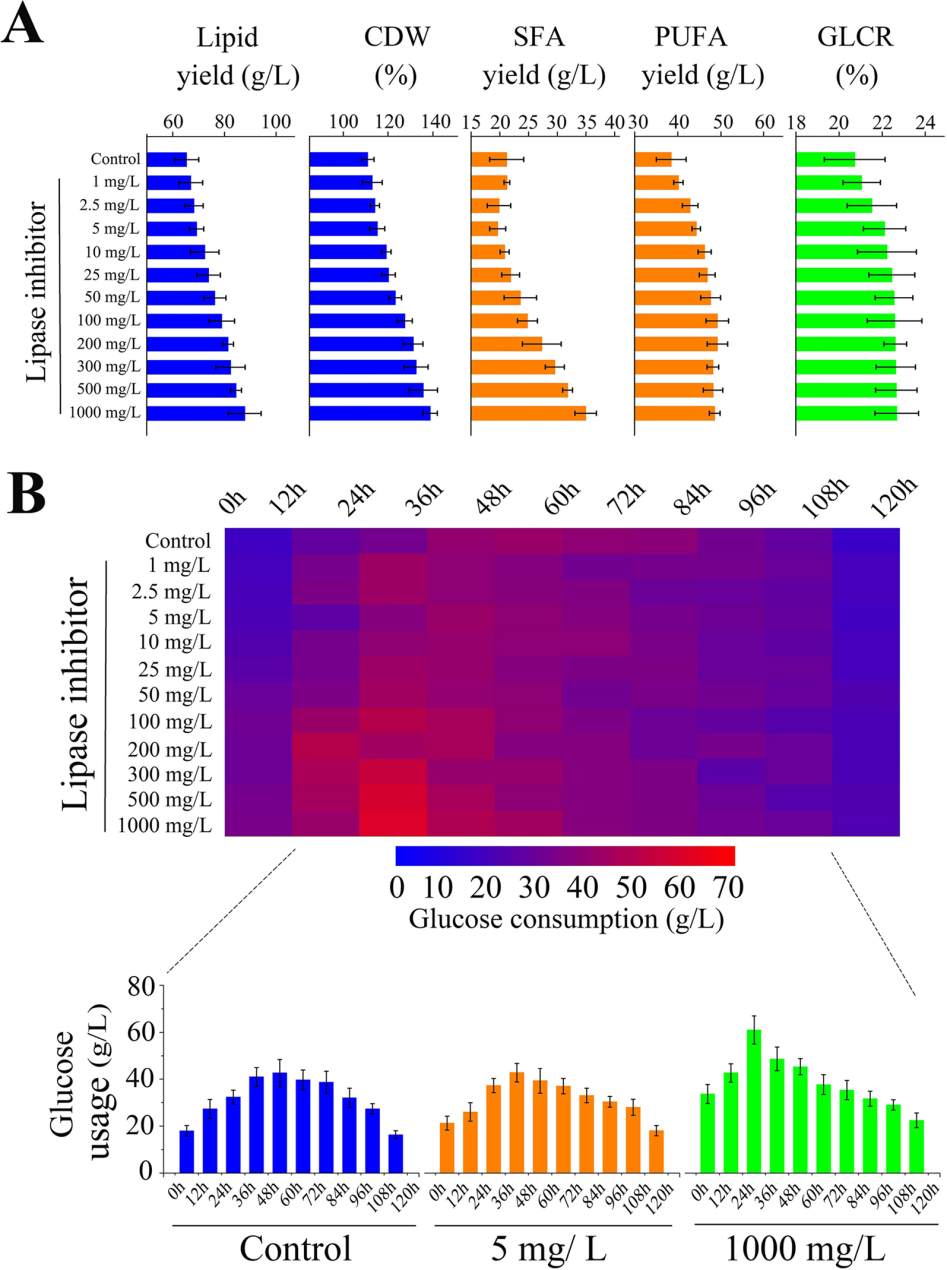
The effect of different lipase inhibitor concentration on the fermentation of *Schizochytrium* sp. HX-308. **A** Changes in Lipid yield, CDW, SFA, PUFA, GLCR and **B** glucose consumption under fermentation conditions in the presence of different concentrations of lipase inhibitors

The screening results for the optimal concentration of orlistat are shown in Fig. 2. It was obvious that the lipid production and CDW of *Schizochytrium* sp. significantly increased with the increase of orlistat concentration (0–1000 mg/L). After 120 h of fermentation, 87.89 g/L lipid production and 138.83 g/L CDW were obtained at a concentration of 1000 mg/L orlistat, which were 1.34 and 1.25 times higher than that of the control, respectively. Furthermore, the addition of lipase inhibitors also had a significant impact on the fatty acid composition of *Schizochytrium* sp. In the concentration range of 0–5 mg/L, the proportion of SFA decreased with an increase in orlistat concentration. Moreover, the proportion of SFA gradually increased when the concentration of orlistat was higher than 5 mg/L, and the proportion of SFA accounted for 39.71% of the total fatty acids (TFAs) with 1000 mg/L orlistat. In addition, the promotion of lipid yield in *Schizochytrium* sp. gradually decreased when the concentration of orlistat exceeded 100 mg/L (Fig. 2A). It illustrates that the GLCR increased as the concentration of orlistat increased, and up to 22.69% at 1000 mg/L orlistat, which was 9.45% higher than that of the control. Not surprisingly, the addition of orlistat accelerated the consumption of glucose by *Schizochytrium* sp., and the total glucose consumption was 1.23 times higher than that of the control when orlistat concentration was 1000 mg/L (Fig. 2B).

**Fig. 3:**
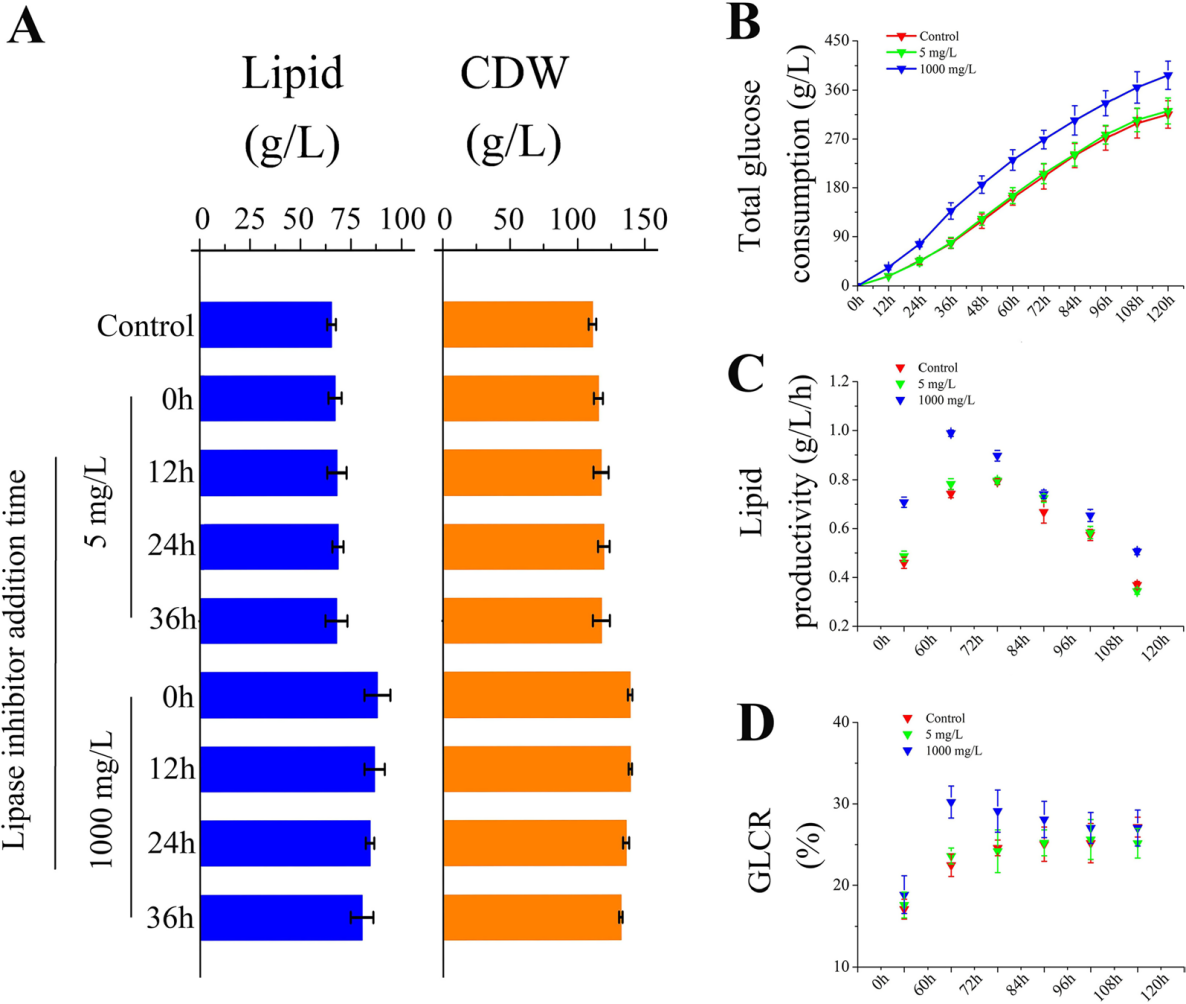
Effect of 5 mg/L and 1000 mg/L lipase inhibitor addition time for **A** Lipid, CDW and **B** total glucose consumption and **C** lipid productivity and **D** GLCR

The addition of chemical regulators at different time points may yield different results [22]. Therefore, 0 h (fermentation start), 12 h (exponential growth period), 24 h (late growth period), and 36 h (rapid lipid accumulation period) were chosen to add the lipase inhibitor with concentrations of 5 mg/L and 1000 mg/L. After 5 days of fermentation, the addition of orlistat at different stages resulted in different changes in lipid productivity (Fig. 3). In the 5 mg/L orlistat treatments, adding orlistat at 24 h yielded 69.24 g/L lipid production, which was 5.94% higher than the control. Additionally, the proportion of PUFAs increased to 68.41%, while that of the control group was only 58.8% (Additional file 1: Fig. S2). The lipid yield in the 1000 mg/L orlistat treatments decreased with the delay in the orlistat supplementation time, and a lipid yield of 87.89 g/L was obtained at 0 h, which was 34% higher than that of the control (Fig. 3A). It was speculated that after low concentrations of orlistat are metabolized by *Schizochytrium* sp., lipase will have a rapid consumption period of lipids, and adding inhibitors at 24 h can delay the rapid consumption of lipids to the later stages of fermentation. Recently, Chang et al. found that TAG lipase may be involved in the preferential hydrolysis of SFAs in the process of lipid turnover [23]. The changes in the ratio of fatty acids during the non-feeding fermentation process also support this conclusion (Additional file 1: Figs. S3, S4), which may explain the experimental phenomenon was observed. Therefore, adding 1000 mg/L lipase inhibitor at 0 h improved lipid accumulation, and adding 5 mg/L lipase inhibitor at 24 h resulted in a higher proportion of PUFAs.

Figure 3B shows that the addition of orlistat led to a significantly higher consumption of glucose by *Schizochytrium* sp. than the control. Moreover, as shown in Fig. 3C, the lipid productivity increased with culture time in the three groups, with the 1000 mg/L orlistat treatment reaching its maximum at 60–72 h, whereas the maximum lipid productivity of the control was at 72–84 h. Compared with the control, the orlistat treatments exhibited an overall higher lipid productivity during the entire fermentation period. Even at the end of fermentation, the lipid productivity reached 0.49 g/L/h in the 1000 mg/L orlistat treatment, which was about 1.3-fold higher than in the control. In addition, orlistat resulted in a change in GLCR, which increased by 21.88% compared with the control at a concentration of 1000 mg/L orlistat in the middle stage of fermentation (60–96 h). In a word, by adding the lipase inhibitor orlistat, the lipid productivity and GLCR of *Schizochytrium* sp. are improved, which greatly saves the substrate cost and time cost of producing microbial lipids (Table 1).

**Table 1:** Some methods to improve the productivity and GLCR of Thraustochytrids

Strain	Strategy	Lipid productivity (g/L/h)	GLCR (%)	References
*Schizochytrium* sp. HX-308	Adaptive evolution by continuous high oxygen	0.471 ± 0.02	14.14 ± 0.5	[24]
*Schizochytrium* sp. HX-308	Adaptive evolution under high salinity stress	0.528 ± 0.03	20.05 ± 0.4	[25]
*Thraustochytrium* sp. T01	Organic acids/phosphate salt addition	0.128 ± 0.01	–	[26]
*Aurantiochytrium* sp. SW1	Kinetin, jasmonic acid, gibberellic acid addition	0.154 ± 0.01	–	[27]
*Schizochytrium* sp.	Malonate addition	0.231 ± 0.02	–	[28]
*Schizochytrium* sp.	Ascorbic acid as elicitor	0.218 ± 0.02	21.81 ± 0.4	[29]
*Aurantiochytrium* sp. SD116	Overexpression of G6PDH	0.055 ± 0.01	11.01 ± 0.2	[30]
*Schizochytrium* sp. ATCC20888	Heterologous overexpression of multiple-genes	0.112 ± 0.01	18.75 ± 0.4	[31]
*Schizochytrium* sp. TIO1101	Overexpression of ACS gene	0.074 ± 0.01	22.33 ± 0.6	[32]
*Schizochytrium* sp. HX-308	Lipase inhibitor addition	0.733 ± 0.02	22.69 ± 0.6	This study

### Effects of lipase inhibitors on cellular antioxidant activity

**Fig. 4:**
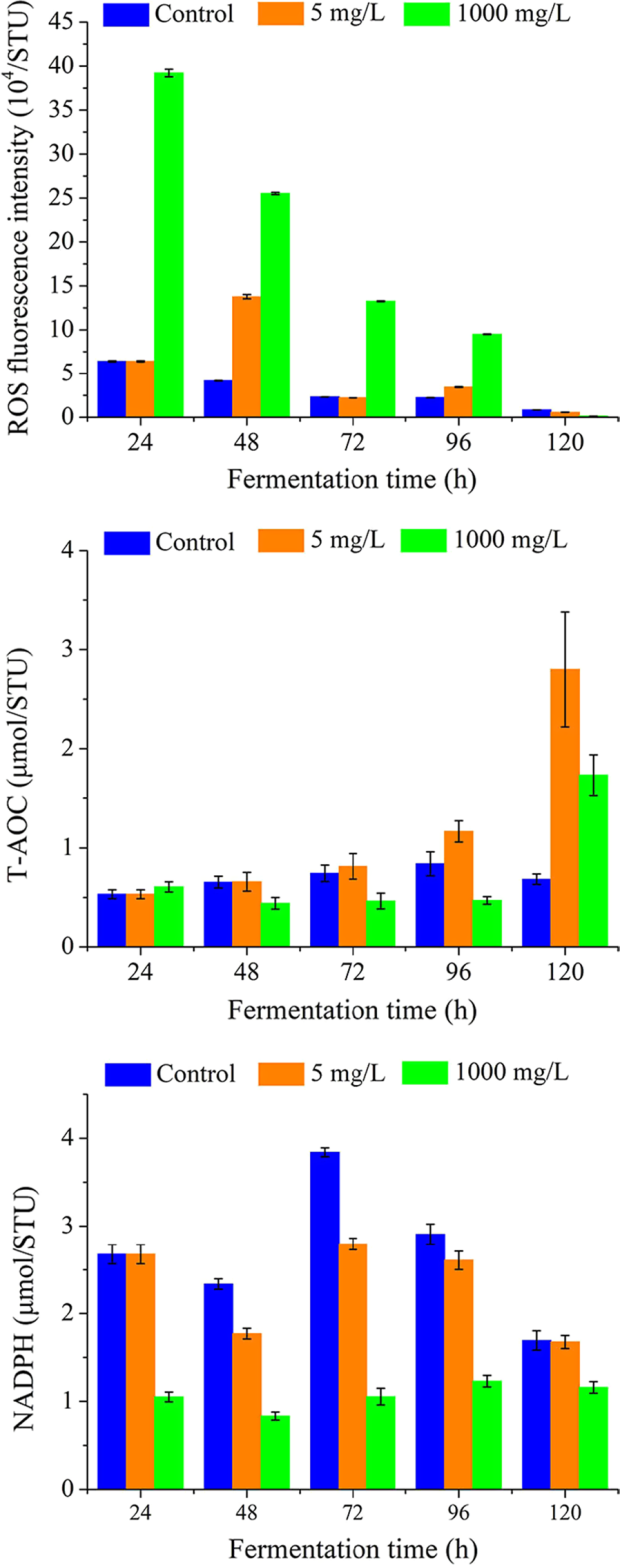
Comparison of the ROS, T-AOC and NADPH content of *Schizochytrium* sp. HS-308 in the presence of orlistat and normal conditions

It was observed that the addition of orlistat caused a significant increase of ROS in *Schizochytrium* sp. (Fig. 4A). In the 1000 mg/L orlistat group, the level of ROS in the cells of the 24 h was 6.1 times that of the control, and at 24–96 h, the ROS in the 1000 mg/L orlistat group was much higher than that of the control. When 5 mg/L orlistat was added after 24 h of fermentation, the ROS increased significantly at 48 h, reaching 3.3 times that of the control group, and then dropped rapidly. The T-AOC of *Schizochytrium* sp. under normal fermentation conditions gradually increased during the fermentation process, and reached the maximum at 96 h (Fig. 4B). Interestingly, the T-AOC of the 5 mg/L orlistat addition group also gradually increased, but the T-AOC level exceeded the control group at 72 h, and reached the maximum at 120 h. Meanwhile, the T-AOC of the 1000 mg/L orlistat added group was at a low level from 24 to 96 h, but increased rapidly at 120 h, and almost three times that of the control group. NADPH is an essential reducing power for fatty acid synthesis. It was observed that the NADPH level of the control group was always higher than the orlistat addition group from 24 to 120 h (Fig. 4C).

### Dynamic expression of genes associated with the lipase inhibitor

**Fig. 5:**
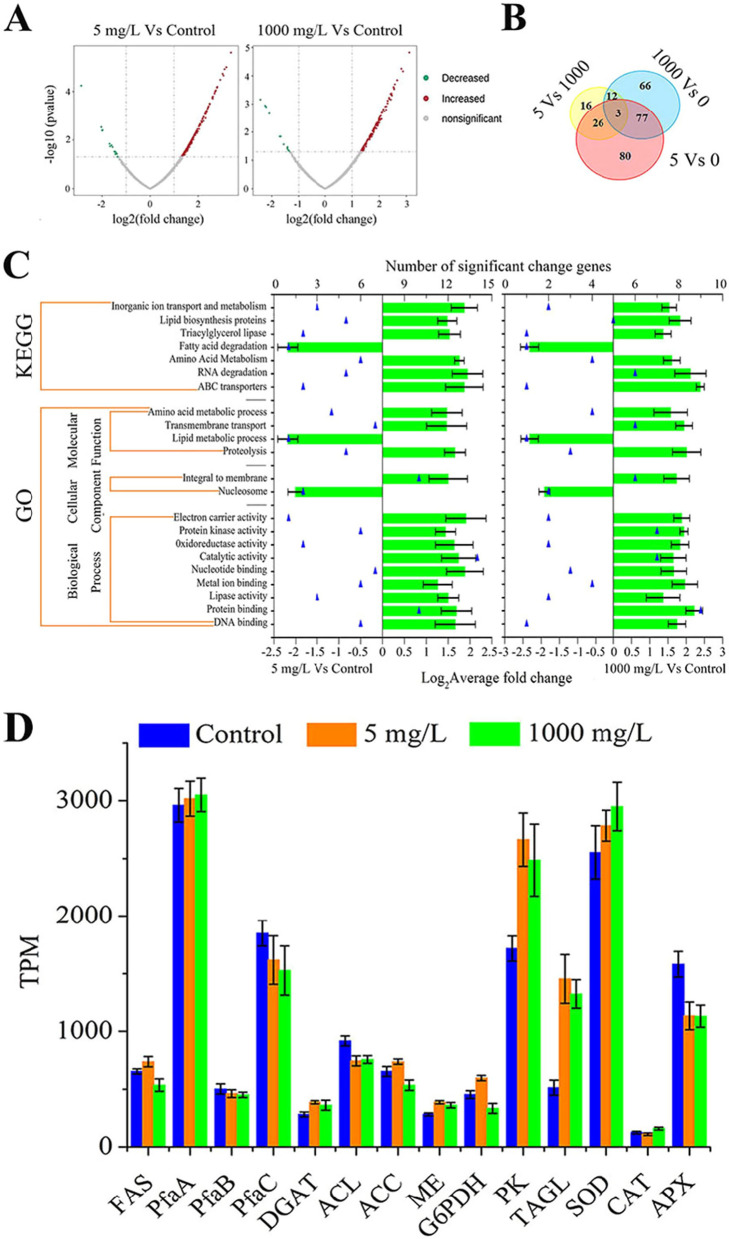
**A** Volcano plots showing p-values (− log10) vs. feature ratio of (log2), 5 mg/L vs. Control and 1000 mg/L vs. Control. **B** Venn diagram showing the unique and overlapping differentially abundant features in different orlistat concentration. **C** Functional analysis (including KEGG pathways and GO classes for gene classification) of gene with significantly changed induced by orlistat under 5 mg/L and 1000 mg/L. **D** Analysis of the transcription level of key enzyme genes of *Schizochytrium* in orlistat and normal conditions at 72 h. The three subunits of the PKS gene, include PfaA, PfaB and PfaC; DGAT gene: diacylglycerol acyltransferase gene; SOD gene: superoxide dismutase gene; CAT gene: catalase gene; APX gene: ascorbate peroxidase gene

During the oscillation, significant changes were detected in the expression of genes in the orlistat treatment groups, particularly, the transcription of genes responding to fatty acid metabolism at 72 h (Fig. 5). As shown in Fig. 5A, approximately 7900 genes from *Schizochytrium* sp. were included in the statistical analysis. Regarding DEGs, there were 170 and 145 upregulated genes, and 16 and 13 downregulated genes in the 5 mg/L and 1000 mg/L orlistat treatment groups compared with the control, respectively. Orlistat addition induced modulations of 264 significant DEGs, of which 80 showed overlap in the 5 mg/L and 1000 mg/L orlistat treatments. In contrast, 106 and 78 DEGs were expressed exclusively in the 5 mg/L and 1000 mg/L orlistat treatments, respectively (Fig. 5B), suggesting drastic metabolic reorganization to combat the stress of orlistat addition and enhance the chance of survival.

Under the two orlistat treatments, the average transcriptional level of ABC transporters that require ATP consumption increased by 3.85 and 4.35 times, respectively. At the same time, electron carrier activity and oxidoreductase activity-related gene transcription levels were also significantly upregulated (Fig. 5C). In addition, five key genes related to fatty acid synthesis and lipid accumulation (i.e., FAS, PfaA, PfaB, PfaC and DGAT), two key genes related to the accumulation and utilization of acetyl-CoA (i.e., ACC and ACL), two key genes related to NADPH supply (i.e., ME and G6PDH), one key gene involved in glycolysis pathway (i.e., PK), one gene related to TAG hydrolysis (i.e., TAGL), and three key genes related to the oxidative defense system (i.e., SOD, CAT and APX) were analyzed by the transcription levels at 72 h. Compared with normal culture, the transcription levels of PK and TAGL genes were significantly upregulated in the orlistat addition group. However, the presence of high concentrations of orlistat inhibited TAGL from functioning, thus leading to the accumulation of lipids. Interestingly, five genes related to fatty acid biosynthesis (FAS, PfaA, PfaB, PfaC and DGAT) and the two main genes produced by NADPH (G6PDH and ME) were no significant change, which also verified that the accumulation of lipids was originated from the inhibitory effect of orlistat on lipase.

### Metabolite profiling of *Schizochytrium* sp. HX-308 with lipase inhibitor

**Fig. 6:**
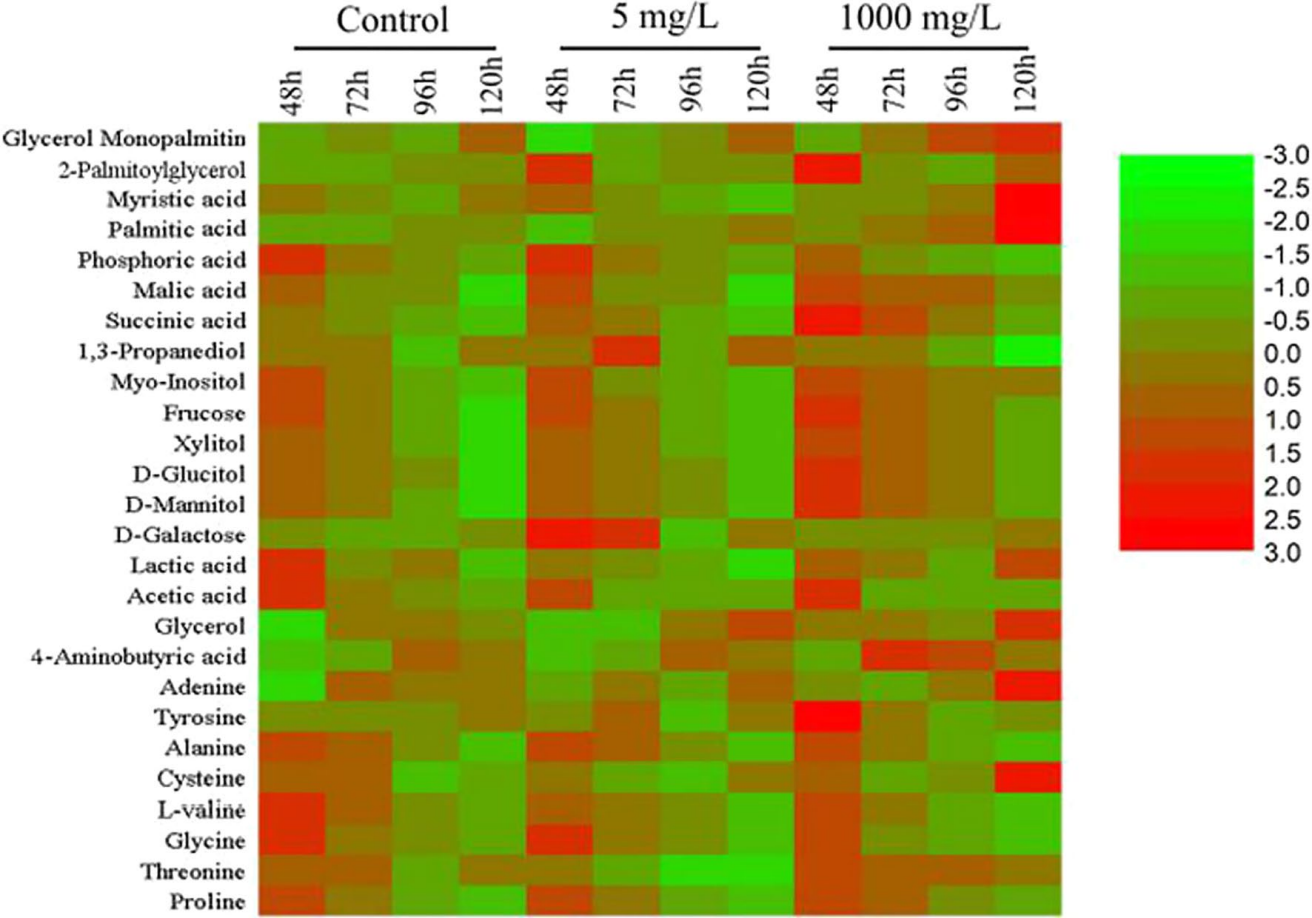
Heat map of the significantly changed metabolites in *Schizochytrium* sp. HX-308 induced by orlistat under 5 mg/L and 1000 mg/L

Compared with the control treatment, glucose consumption increased over time in the 1000 mg/L orlistat treatment group, and the fructose, mannitol, and xylitol concentrations also increased (Fig. 6). In addition, the galactose and sorbitol concentrations increased. The changes in the fructose, mannitol, and galactose concentrations indicated that the addition of orlistat can accelerate the rate of glucose metabolism and change intracellular glucose metabolism to prevent and deal with the possible external adverse environment. Additionally, glycine concentration converted from 3-phosphoglycerate and alanine converted from pyruvate changed significantly compared with the control. The amino acid content remained high at 48 h and then declined in the later fermentation stages (Additional file 1: Fig. S5). The TCA cycle is the main source of ATP for lipid accumulation in *Schizochytrium* sp., and succinic acid is the main metabolite in the TCA cycle. It showed that the succinic acid content in the orlistat treatment groups was higher than that in the control group. The increase in the succinic acid content indicates that the carbon metabolic flow of the TCA cycle was active during the fermentation process, which implied that the addition of orlistat might cause the migration of the metabolic flow, resulting in a large amount of acetyl-CoA entering the TCA cycle to produce more ATP for the growth of *Schizochytrium* sp. and fatty acid accumulation, which was consistent with the transcriptome analysis. At the same time, the proline and 4-aminobutyric acid concentrations increased compared with the control, especially 4-aminobutyric acid, which was low before 48 h. Upon entering the lipid accumulation period, the concentration of 4-aminobutyric acid increased rapidly. The control reached its peak at 96 h, which was 1.75 times higher than the initial intracellular concentration, while the peak in the 1000 mg/L orlistat treatment group was earlier (Additional file 1: Fig. S5).

The addition of orlistat reduced the phosphoric acid concentration in the metabolites of *Schizochytrium* sp., but the concentration of inositol increased significantly. When *Schizochytrium* sp. entered the period of lipid accumulation, the enhancement of the GABA metabolic pathway could supplement the deficiency of NADH and maintain intracellular reactive oxygen species levels, which is beneficial for lipid accumulation, while proline and myo-inositol played an important biological function for cells to adapt to environmental pressure. During the fermentation process, the phosphoric acid concentration also changed significantly.

In addition, the fermentation conditions of orlistat were tested in Thraustochytrid *Aurantiochytrium*. Unlike *Schizochytrium* sp., 5 mg/L orlistat obviously promoted *Aurantiochytrium* sp. to accumulate saturated fatty acids, while 1000 mg/L orlistat had no obvious effect on the fatty acid composition of *Aurantiochytrium* sp., which might be the different types of lipase in *Aurantiochytrium* sp. and *Schizochytrium* sp. (Additional file 1: Figs. S6, S7). After the 120 h fermentation, *Aurantiochytrium* sp. lipid productivity and GLCR increased by 11.18% and 4.59%, respectively (Additional file 1: Fig. S8). The result proved that the applicability of orlistat fermentation conditions for the production of microbial lipids by thraustochytrids.

## Discussion

**Fig. 7:**
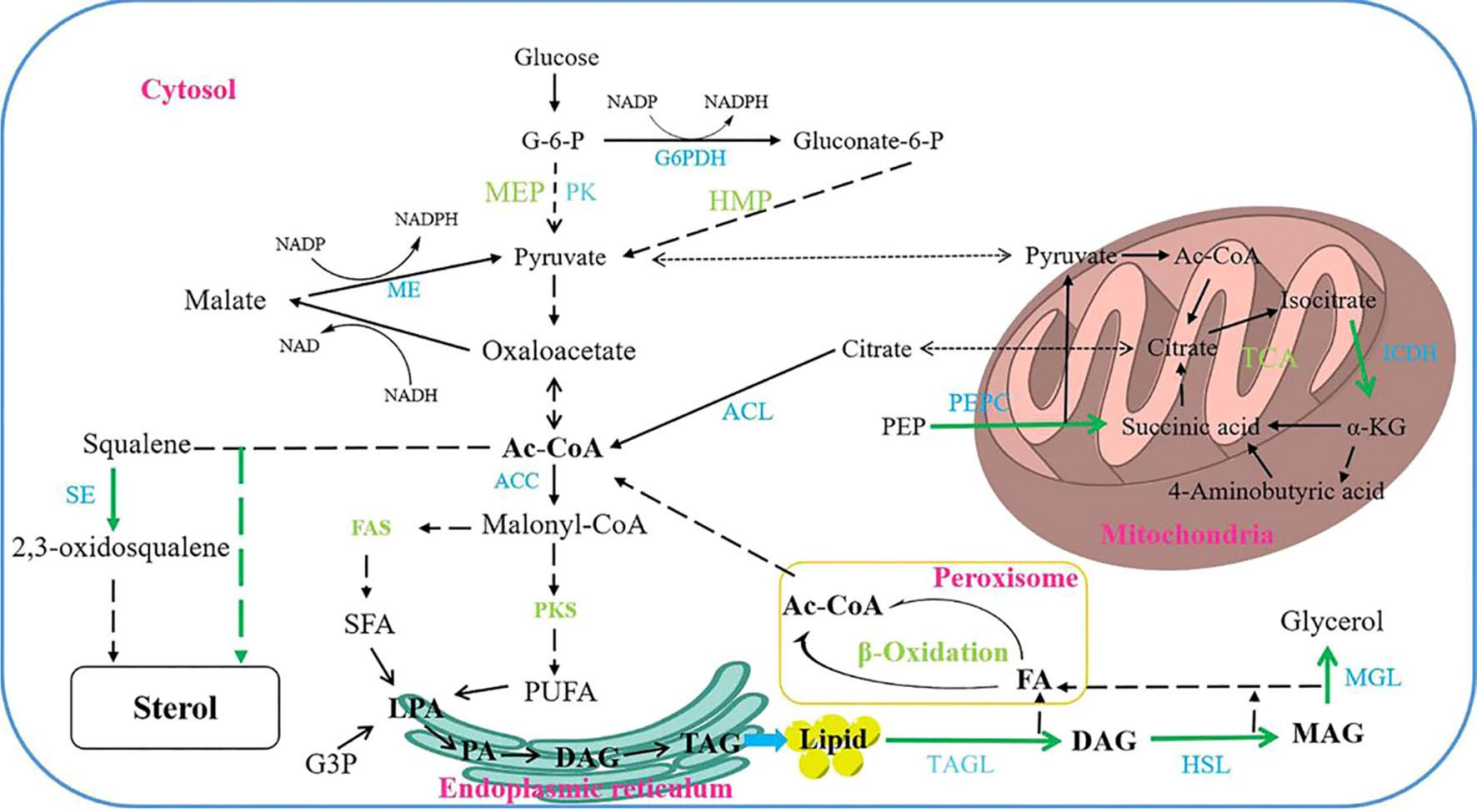
Schematic map of metabolic pathways on lipid accumulation in *Schizochytrium* sp. HX-308. The green arrows represent the metabolic pathways of using chemical modulators. G-6-P, glucose-6-phosphoric; G3P, glucose-3-phosphoric; PK, pyruvate kinase; LPA, lysophosphatidic acid; PA, phosphatidic acid; DAG, diacylglycerol; FA, fatty acid; α-KG, α-ketoglutarate; PEP, phosphoenolpyruvate; G6PDH, glucose-6-phosphate; SE, squalene epoxidase; ACC, Acetyl-CoA carboxylase; ACL, ATP-citrate lyase; PEPC, phosphoenolpyruvate carboxylase; ICDH, isocitrate dehydrogenase; TAGL, triacylglycerols lipase; HSL, hormone-sensitive lipase; MGL, monoacylglycerol lipase; ME: malic enzyme; MEP, methylerythritol-phosphate; HMP, Hexose Monophophate Pathway; TCA, tricarboxylic acid cycle

Sterol, squalene and lipids are the main metabolites of *Schizochytrium* sp*.* [33], the inhibition of the squalene epoxidation pathway and sterol biosynthesis pathway is theoretically feasible to provide acetyl-CoA for lipid synthesis. The inhibition of PEPC and ICDH activity has a positive effect on lipid accumulation in other lipid-producing microorganisms and may increase lipid accumulation in *Schizochytrium* sp*.* Similarly, the degradation and utilization of TAGs by lipase is an obstacle to lipid accumulation, especially in the middle and late stages of fermentation (Fig. 7). Thus, PEPC may convert PEP into succinic acid, and the accumulation of succinic acid can enhance the supply of ATP. The addition of quinoxaline to inhibit this process was not conducive for the growth of *Schizochytrium* sp., and inhibition of sterols production also had an adverse effect on lipid accumulation. Lipids are important energy substances, and the use of lipids first requires lipase is required to hydrolyze TAG into free fatty acids, which releases energy through the β-oxidation pathway [33]. It is well documented that the knockout of lipase can significantly promote the accumulation of lipids in microalgae [19]. Orlistat is a potent lipase inhibitor, which achieves the effect of lipid accumulation by inhibiting the degradation of TAG by lipase [34]. Therefore, the addition of lipase inhibitors may improve the lipid yield and GLCR of *Schizochytrium* sp.

Orlistat's inhibition of lipase function has caused the ability to use lipids to maintain normal life activities to be greatly damaged, so *Schizochytrium* sp. strengthens the metabolism of glucose to supplement the loss of ATP and acetyl-CoA caused by the downregulation of fatty acid β-oxidation pathway. At a concentration of 1000 mg/L orlistat, the proportion of SFA accounted for 39.71% of the TFAs. This may be because acetyl-CoA and ATP provided by glucose metabolism are also used to synthesize fatty acids, and the inhibition of TAG lipase function also has a positive effect on the accumulation of SFAs. In addition, compared with the synthesis of PUFAs, SFA synthesis requires less acetyl-CoA through the fatty acid synthase (FAS) pathway, and requiring less ATP to activate acetyl-CoA is another possible reason (Additional file 1: Fig. S9). It is also worth noting that the addition of orlistat improved the GLCR, and this change was gratifying, because glucose is the main substrate cost used in lipid production by *Schizochytrium* sp. [34]. Zhang et al. found that *thraustochytrids* transfer the main energy metabolism of terpenoid biosynthesis from carbohydrates to lipid β-oxidation at 60 h, so the addition of lipase inhibitors inhibits this process and leads to an increase in GLCR [35].

In addition, oxygen-consuming microorganisms can transfer the generated hydrogen and electrons to oxygen through electron transfer and oxidative phosphorylation, so that organic matter is oxidized and decomposed to produce carbon dioxide and water and provide energy for their own growth and metabolism [36]. At the same time, cells will inevitably form various types of ROS through different mechanisms, but the accelerated production of ROS has a toxic effect on cell viability. Therefore, the addition of lipase inhibitors promotes the rapid accumulation of lipids and resulting in a rapid depletion of nutrients, therefore, leads to a large increase in ROS [25]. Microorganisms develop a series of mechanisms to remove ROS, such as various antioxidants and antioxidant enzyme systems, to reduce the toxic effects of ROS on cells, which can be evaluated by T-AOC. However, the T-AOC level of the orlistat supplementation group gradually exceeded the control group only in the late fermentation period, which may be the temporary failure of the T-AOC system due to the high ROS level in the early fermentation period. In general, reducing power is necessary for cell growth and metabolism. The addition of orlistat inhibits lipid degradation, but the enhanced glycolysis pathway provides more acetyl-CoA to enter the fatty acid synthesis pathway, thus leading to excessive consumption of NADPH.

Consistent with our previous speculation, the ATP gap caused by the downregulation of the fatty acid β*-*oxidation pathway is supplemented by other metabolic pathways, but the fatty acid synthesis pathway still consumes a large amount of ATP. The increase in the transcription level of the ABC transporter indirectly confirmed the increase in ATP synthesis [37]. The upregulation of genes related to electron carrier activity and oxidoreductase activity is not only conducive to the supply of NADPH, but also is conducive to the accumulation of lipids by improving the antioxidant capacity of *Schizochytrium* sp. [29]. The increase in PK gene transcription level means that *Schizochytrium* sp. uses more glucose, which provides more ATP and acetyl-CoA for fatty acid synthesis; while the increase in TAGL gene transcription level means that more lipases that hydrolyze TAG are produced, which is unfavorable for lipid accumulation [38]. This conclusion can also be confirmed by the downregulation of gene expression levels related to Fatty acid degradation pathway. Microorganisms have a complete antioxidant enzyme system to remove active oxygen generated by life activities. These systems are mainly composed of SOD, CAT and APX [39]. Among them, SOD can catalyze the disproportionation of extremely toxic superoxide anions into oxygen and hydrogen peroxide [40]. Then, under the catalysis of CAT or APX, hydrogen peroxide is removed to generate H_2_O and O_2_. Therefore, the SOD gene showed a higher transcription level due to the high level of ROS accumulation in the orlistat addition group.

The carbon source is the basic substance used by microorganisms to maintain cell growth and survival. Glucose, the most common carbon source, is a key component of *Schizochytrium* sp. metabolism. The high consumption of glucose caused by the addition of orlistat not only accumulates lipids, but also intensifies the flow of intracellular glucose metabolism to other metabolic pathways. Glucose meets the needs of cell growth but is also involved in the synthesis of polysaccharides and their derivatives. Amino acids are necessary for the metabolism of carbon and nitrogen, and are important precursors for the synthesis of proteins, nucleotides, and other cellular components [41]. They play important roles in the regulation of cell growth and metabolism, and maintaining cell physiological functions. Phosphoric acid is an important component of the phospholipid bilayer of cell membranes and organelle membranes, while myo-inositol has a variety of physiological functions; it not only stores carbohydrates and NADPH, but also acts as a coenzyme regulator [42]. 4-aminobutyric acid is the core substance of the γ-aminobutyric acid (GABA) metabolic pathway and cannot be ignored. The GABA metabolic pathway is a pathway that produces NADH energy [22, 43]. Ren et al. had reported that under phosphorus-limited conditions, ME and G6PDH maintain high activity levels and provide a large amount of NADPH [44]. Therefore, the reduction of phosphoric acid may also increase the activity of certain enzymes in *Schizochytrium* sp. thus promote the accumulation of fatty acids.

## Conclusion

The green synthesis of lipids through microbial fermentation is attractive, but it is difficult to carry out because of its high costs and low productivity. In this study, we added 1000 mg/L lipase inhibitor before the start of fermentation to obtain a lipid yield of 87.89 g/L, which was 34.39% higher than that of the control, while the GLCR, PUFA and SFA yield were increased 9.45%, 64.52% and 24.04%, respectively. And orlistat fermentation conditions also have been successful in Thraustochytrid *Aurantiochytrium*. The transcription and metabolomics analysis showed that the inhibition of the TAG degradation pathway and the enhancement of energy supply are key to increasing the lipid accumulation. In the presence of lipase inhibitors, the DEGs related to the fatty acid degradation pathway in *Schizochytrium* sp. were significantly downregulated, and the demand for glucose was significantly increased. From the perspective of industrial development, our work is competitive, because it is a simple, effective approach to increase lipid productivity and GLCR at the same time. Additionally, our work offers an insight into the production of other microbial lipids and the design of an engineered *Schizochytrium* sp. with higher lipid productivity by genetic engineering in the future.

## Materials and methods

### Chemicals and screening standard

Quinoxalin and hexaconazole were purchased from Aladdin (Shanghai, China), and terbinafine hydrochloride, vorasidenib, and orlistat were purchased from Macklin (Shanghai, China). These chemicals were of analytical purity and were dissolved in ethanol. Adonitol, Methoxyamine hydrochloride and pyridine were purchased from Macklin (Shanghai, China), and N-methyl-N-(trimethylsilyl) trifluoroacetamide was purchased from Sigma (Burlington, USA). All samples were prepared in glass vials and stored at 4 °C. Quinoxalin (10 mg/L), hexaconazole (10 mg/L), terbinafine (10 mg/L), vorasidenib (0.1 mg/L), and orlistat (10 mg/L) were added to the fermentation medium at the beginning of fermentation.

The lipid yield, GLCR and fatty acids content were used as indicators to evaluate their performance. The GLCR was determined as follows:

$$ {\text{GLCR}} = \frac{{{\text{Total}}\;{\text{lipid}}\;{\text{production}}}}{{{\text{Total}}\;{\text{glucose}}\;{\text{usage}}}}\;\left( {{{{\text{wt}}} \mathord{\left/ {\vphantom {{{\text{wt}}} {{\text{wt}}\% }}} \right. \kern-\nulldelimiterspace} {{\text{wt}}\% }}} \right). $$

### Microorganism and culture conditions

*Schizochytrium* sp. HX-308 (CCTCC M 209059) was isolated from seawater and stored in the China Center for Type Culture Collection. The strain was preserved in 20% (v/v) glycerol at − 80 °C. The seed culture medium and culture conditions were the same as those used in a previous study [45]. The seeds of *Schizochytrium* sp. cells were cultured for three passages and then transferred to 500 ml shake flasks containing 100 mL of the medium at a final concentration of 10% for the batch culture. Finally, *Schizochytrium* sp. was maintained in artificial seawater with 100 g/L glucose and 5 g/L yeast extract at 28 °C under constant orbital shaking at 170 rpm.

The artificial seawater contained: 20 g/L monosodium glutamate, 10 g/L Na_2_SO_4_, 4.8 g/L KH_2_PO_4_, 4.8 g/L MgSO_4_·7H_2_O, 2.8 g/L (NH4)_2_SO, 1 g/L KCl, and 0.2 g/L CaCl_2_. The trace element solution contained 6 mg/L Na_2_EDTA, 8 mg/L MnCl_2_·4H_2_O, 0.8 mg/L ZnSO_4_, 0.29 mg/L FeSO_4_, 0.01 mg/L CoCl_2_·6H_2_O, 0.6 mg/L CuSO_4_·5H_2_O, 0.06 mg/L NiSO_4_·6H_2_O, and 0.1 mg/L Na_2_MoO_4_·2H_2_O. In addition, a vitamin B mixture (20 mg/L) was also added to the fermentation medium.

### Cell dry weight, total lipids, and fatty acid methyl ester analysis

The cell dry weight (CDW) was determined gravimetrically by filtering 10 mL of the samples using a centrifuge for 5 min at 6500*g*. The sample was dried for 48 h at 60 °C by transferring the cells to dried and weighed filter paper. During the drying process, the weight was recorded repeatedly until the mass remained stable. The fermentation broth (50 mL) was first high-temperature (50 °C) enzymatic hydrolysis to break the wall of the *Schizochytrium* sp. HX-308 cells, inactivate the wall-breaking enzyme with ethanol, and perform rotary steaming after extraction with n-hexane (45–50 °C) to get lipids. Finally, the total lipids were calculated by weighing.

Fatty acid methyl esters were prepared by adding 2 mL of 1 M potassium hydroxide-methanol to a glass tube containing 0.2 g of dried cells. The glass tube was heated in a 60 °C water bath for 30 min and then cooled to room temperature. At this time, 2 mL of boron trifluoride ether (boron trifluoride:ether = 3:7, v/v) was added and the solution mixed, and then it was placed in a 60 °C water bath and heated again for 10 min. When the tube was cooled to room temperature, 2 mL of saturated sodium chloride aqueous solution was added, mixed well, and then 3 mL of n-hexane was added, mixed well, and allowed to stand until the layers separated. The upper n-hexane phase was taken through the membrane and gas chromatography (GC) detection (GC-2030, Shimadzu, Japan); the GC-related settings and procedures are as described above [24]. All tests were conducted in triplicate, and the results were expressed as the mean ± standard deviation.

### Evaluation of anti-oxidation ability

The relative ROS content of the samples were evaluated by the fluorescent probe 2′,7′-dichlorodihydrofluorescein diacetate (DCFH-DA) [46]. DCFH-DA fluorescent probe was dissolved in dimethyl sulfoxide to 1 mM, and store it at – 20 °C for later use, then added to the cell culture in a 1:100 ratio, and incubated at 30 °C, 170 rpm and dark for 20 min, so that the fluorescent probe can fully enter the cell. The cell samples were washed twice with PBS buffer to sufficiently remove extracellular DCFH-DA, and then dissolve the cell sample in PBS. Finally, the relative content of intracellular ROS was measured by multifunctional microplate reader (Gene Company Limited, China) with excitation at 488 nm and emission at 525 nm. The T-AOC of *Schizochytrium* sp. was determined by Micro Total Antioxidant Capacity Assay Kit (Solarbio, China) according to the manufacturer’s instructions. NADPH content was determined using the NADP/NADPH Quantification Kit (Beyotime, China) following the instruction of the manufacturer.

One standard unit (STU) is defined as the result produced by the reaction of 1 mL of OD_600_ = 1 bacterial liquid.

### Transcriptomic analysis

High-resolution genome-wide transcription analysis was performed using RNA‐Seq at Hangzhou Kaitai Biotechnology Co., Ltd (Hangzhou, Zhejiang, China). Raw reads were filtered to remove adapters and low-quality reads, and the remaining reads were mapped to the reference genome, *Schizochytrium* sp. HX-308, for transcript information. Gene expression was normalized by transcripts per kilobase per million mapped reads (TPM), and the number of transcripts per kilobase per million mapped reads was calculated as follows:

$$ {\text{TPM}}_{i} = \frac{{N_{i} }}{{L_{i} }} \cdot \left( {\frac{1}{{\sum\nolimits_{j} {\frac{{N_{j} }}{{L_{j} }}} }}} \right) \cdot 10^{6} , $$

where *N*_i_ is the number of reads mapped to the target gene, *L*_i_ is the total length of the exons of the target gene, *N*_j_ is the number of reads mapped to any gene in the sample, and *L*_j_ is the sum of the lengths of the exons of any gene in the sample.

Differentially expressed genes (DEGs) were defined as follows: the sum of mapping reads ≥ 10 in the two samples, satisfying |log_2_(fold change)|> 1; a false discovery rate (FDR) correction was performed on the *p*-value to obtain the q-value, and at the same time must satisfy the *p*-value ≤ 0.05 and *q*-value ≤ 0.05.

### Derivatization and gas chromatography–mass spectrometry analysis

To investigate the mechanism by which orlistat increased lipid production by *Schizochytrium* sp. HX-308, a gas chromatography–mass spectrometry (GC–MS) method combined with multivariate analyses was used to determine the changes in metabolites in the presence of different concentrations of orlistat. The sample derivatization process was in accordance with a previously reported method [15], with some modifications. The above sample was mixed with 25 μL of an internal standard (Adonitol, 0.2 mg/mL) and dried with a nitrogen blower. Methoxyamine hydrochloride (40 μl) in pyridine (20 mg/mL) was added to the dried sample and incubated at 40 °C for 1 h. Then, the sample was silylated for 2 h at 30 °C by adding 60 μl of N-methyl-N-(trimethylsilyl) trifluoroacetamide and vortex mixing for 60 s.

The GC–MS system included a GC (GC-2030, Shimadzu, Japan) with a DB-5MS capillary column (parameter: 60 m × 0.25 mm × 0.25 µm, Agilent, USA) and mass spectrometer (GCMS-QP2020 NX, Shimadzu, Japan). Ionization was performed using an electrospray ionization source operating in either the positive or negative ionization mode, and helium was used for collision-induced dissociation at a flow rate of 1 mL/min (constant flow). The GC temperature programming was set at 70 °C for 2 min, then increased to 300 °C in increments of 5 °C/min and held for 3 min. The temperatures of the injection, transfer line, and ion source were 250, 280, and 230 °C, respectively. The mass scan range was 50–800 m/z.

## Supplementary Information

**Additional file 1. **Additional figures and table.

## Data Availability

All data generated or analysed during this study are included in this published article and its additional information files.

## Abbreviations

ACC: Acetyl-CoA carboxylase
ACP: Acyl carrier protein
AT: Acyltransferase
ATP: Adenosine triphosphate
APX: Ascorbate peroxidase
ACL: ATP-citrate lyase
CAT: Catalase
CDW: Cell dry weight
Cys: Cysteine
DHA: Docosahexaenoic acid
DPA: Docosapentaenoic acid
DH: Dehydratase
DAG: Diacylglycerol
DGAT: Diacylglycerol acyltransferase
DCFH-DA: 2′,7′-Dichlorodihydrofluorescein diacetate
ER: Enoylreductase
EPA: Eicosapentaenoic acid
FA: Fatty acid
FAS: Fatty acid synthase
GC: Gas chromatography
GC–MS: Gas chromatography–mass spectrometry
GLCR: Glucose-to-lipid conversion rate
G3P: Glucose-3-phosphoric
G-6-P: Glucose-6-phosphoric
G6PDH: Glucose-6-phosphate
HMP: Hexose Monophophate Pathway
HSL: Hormone-sensitive lipase
ICDH: Isocitrate dehydrogenase
α-KG: α-Ketoglutarate
KR: Ketoreductase
KS: Ketosynthase
LPA: Lysophosphatidic acid
ME: Malic enzyme
MEP: Methylerythritol-phosphate
MGL: Monoacylglycerol lipase
NADPH: Nicotinamide adenine dinucleotide
PA: Phosphatidic acid
PEPC: Phosphoenolpyruvate carboxylase
PEP: Phosphoenolpyruvate
PKS: Polyketide synthase
PUFAs: Polyunsaturated fatty acids
PK: Pyruvate kinase
ROS: Reactive oxygen species
SFAs: Saturated fatty acids
SE: Squalene epoxidase
SOD: Superoxide dismutase
T-AOC: Total antioxidant capacity
TFAs: Total fatty acids
TPM: Transcripts per kilobase per million mapped reads
TAG: Triacylglycerol
TAGL: Triacylglycerols lipase
TCA: Tricarboxylic acid cycle

## References

[1] Morabito C, Bournaud C, Maës C, Schuler M, Rébeillé F (2019). The lipid metabolism in *thraustochytrids*. Prog Lipid Res.

[2] Weill P, PlissoNn Eau C, Legrand P, Rioux V, Thibault R (2020). May omega-3 fatty acid dietary supplementation help reduce severe complications in Covid-19 patients?. Biochimie.

[3] Sohedein M, Wan WM, Ilham Z, Babadi AA, Siew-Moi P (2020). Vital parameters for biomass, lipid, and carotenoid production of *thraustochytrids*. J Appl Phycol.

[4] Patel A, Karageorgou D, Rova E, Katapodis P, Matsakas L (2020). An overview of potential oleaginous microorganisms and their role in biodiesel and omega-3 fatty acid-based industries. Microorganisms.

[5] Nerantzis ME (2013). Microalgae for high-value compounds and biofuels production: a review with focus on cultivation under stress conditions. Biotechnol Adv.

[6] Chen B, Wan C, Mehmood MA, Chang JS, Bai F, Zhao X (2017). Manipulating environmental stresses and stress tolerance of microalgae for enhanced production of lipids and value-added products—a review. Bioresour Technol.

[7] Jakobsen AN, Aasen IM, Josefsen KD, Strm AR (2008). Accumulation of docosahexaenoic acid-rich lipid in thraustochytrid *Aurantiochytrium* sp. strain T66: effects of N and P starvation and O2 limitation. Appl Microbiol Biotechnol.

[8] Huang TY, Lu WC, Chu IM (2012). A fermentation strategy for producing docosahexaenoic acid in *Aurantiochytrium* limacinum SR21 and increasing C22:6 proportions in total fatty acid. Bioresour Technol.

[9] Yin FW, Zhang YT, Jiang JY, Guo DS, Gao S, Gao Z (2019). Efficient docosahexaenoic acid production by *Schizochytrium* sp. via a two-phase pH control strategy using ammonia and citric acid as pH regulators. Process Biochem.

[10] Kaye Y, Grundman O, Leu S, Zarka A, Zorin B, Didi-Cohen S, Khozin-Goldberg I, Boussiba S (2015). Metabolic engineering toward enhanced LC-PUFA biosynthesis in *Nannochloropsis* oceanica: overexpression of endogenous Δ12 desaturase driven by stress-inducible promoter leads to enhanced deposition of polyunsaturated fatty acids in TAG. Algal Res.

[11] Suen YL, Tang HM, Huang JC, Chen F (2014). Enhanced production of fatty acids and astaxanthin in *Aurantiochytrium* sp. by the expression of vitreoscilla hemoglobin. J Agric Food Chem.

[12] Wang S, Lan C, Wang Z, Wan W, Song X (2020). Optimizing eicosapentaenoic acid production by grafting a heterologous polyketide synthase pathway in the thraustochytrid *Aurantiochytrium*. J Agric Food Chem.

[13] Du F, Wang YZ, Xu YS, Shi TQ, Huang H (2021). Biotechnological production of lipid and terpenoid from *thraustochytrids*. Biotechnol Adv.

[14] Garlick JM, Sturlis SM, Bruno PA, Yates JA, Mapp AK (2021). Norstictic acid is a selective allosteric transcriptional regulator. J Am Chem Soc.

[15] Li Z, Ling X, Zhou H, Meng T, Zeng J, Hang W, Shi Y, He N (2019). Screening chemical modulators of benzoic acid derivatives to improve lipid accumulation in *Schizochytrium* limacinum SR21 with metabolomics analysis. Biotechnol Biofuels.

[16] Ratledge C (2004). Fatty acid biosynthesis in microorganisms being used for single cell oil production. Biochimie.

[17] Cheng Jh, Niu Y, Lu S, Yuan YI (2012). Metabolome analysis reveals ethanolamine as potential marker for improving lipid accumulation of model photosynthetic organisms. J Chem Technol Biotechnol.

[18] Liu ZX, You S, Tang BP, Wang B, Sheng S, Wu FA, Wang J (2019). Inositol as a new enhancer for improving lipid production and accumulation in *Schizochytrium* sp. SR21. Environ Sci Pollut Res.

[19] Trentacoste EM, Shrestha RP, Smith SR, Glé C, Gerwick WH (2013). Metabolic engineering of lipid catabolism increases microalgal lipid accumulation without compromising growth. Proc Natl Acad Sci USA.

[20] Yazawa H, Kumagai H, Uemura H (2012). Characterization of triglyceride lipase genes of fission yeast *Schizosaccharomyces* pombe. Appl Microbiol Biotechnol.

[21] Zan X, Cui F, Sun J, Zhou S, Song Y (2019). Novel dual-functional enzyme Lip10 catalyzes lipase and acyltransferase activities in the oleaginous fungus *Mucor*
*circinelloides*. J Agric Food Chem.

[22] Li Q, Zhao Y, Ding W, Han B, Yu X (2020). Gamma-aminobutyric acid facilitates the simultaneous production of biomass, astaxanthin and lipids in *Haematococcus* pluvialis under salinity and high-light stress conditions. Bioresour Technol.

[23] Chang M, Zhang T, Li L, Lou F, Wang X (2021). Choreography of multiple omics reveals the mechanism of lipid turnover in *Schizochytrium* sp. S31. Algal Res.

[24] Sun XM, Ren LJ, Ji XJ, Chen SL, Huang H (2016). Adaptive evolution of *Schizochytrium* sp. by continuous high oxygen stimulations to enhance docosahexaenoic acid synthesis. Bioresour Technol.

[25] Sun XM, Ren LJ, Bi ZQ, Ji XJ, Zhao QY, He H (2018). Adaptive evolution of microalgae *Schizochytrium* sp. under high salinity stress to alleviate oxidative damage and improve lipid biosynthesis. Bioresour Technol.

[26] Chandrasekaran K, Dhanraj M, Chadha A (2018). Effects of organic and inorganic salts on docosahexaenoic acid (DHA) production by a locally isolated strain of *Thraustochytrium* sp. T01. Prep Biochem Biotechnol.

[27] Nazir Y, Halim H, Prabhakaran P, Ren X, Naz T, Mohamed H, Nosheen S, Mustafa K, Yang W, Hamid AA (2020). Different classes of phytohormones act synergistically to enhance the growth, lipid and DHA biosynthetic capacity of *Aurantiochytrium* sp. SW1. Biomolecules.

[28] Zhao B, Li Y, Li C, Yang H, Wang W (2018). Enhancement of *Schizochytrium* DHA synthesis by plasma mutagenesis aided with malonic acid and zeocin screening. Appl Microbiol Biotechnol.

[29] Ren LJ, Sun XM, Ji XJ, Chen SL, Guo DS, Huang H (2017). Enhancement of docosahexaenoic acid synthesis by manipulation of antioxidant capacity and prevention of oxidative damage in *Schizochytrium* sp. Bioresour Technol.

[30] Cui GZ, Ma Z, Liu YJ, Feng Y, Sun Z, Cheng Y, Song X, Cui Q (2016). Overexpression of glucose-6-phosphate dehydrogenase enhanced the polyunsaturated fatty acid composition of *Aurantiochytrium* sp. SD116. Algal Res.

[31] Han X, Zhao Z, Wen Y, Chen Z (2020). Enhancement of docosahexaenoic acid production by overexpression of ATP-citrate lyase and acetyl-CoA carboxylase in *Schizochytrium* sp. Biotechnol Biofuels.

[32] Yan J, Cheng R, Lin X, You S, Ma Y (2012). Overexpression of acetyl-CoA synthetase increased the biomass and fatty acid proportion in microalga *Schizochytrium*. Appl Microbiol Biotechnol.

[33] Quilodrán B, Hinzpeter I, Hormazabal E, Quiroz A, Shene C (2010). Docosahexaenoic acid (C22:6n–3, DHA) and astaxanthin production by *Thraustochytriidae* sp. AS4-A1 a native strain with high similitude to *Ulkenia* sp.: evaluation of liquid residues from food industry as nutrient sources. Enzyme Microb Technol.

[34] Franz AK, Danielewicz MA, Wong DM, Anderson LA, Boothe JR (2013). Phenotypic screening with oleaginous microalgae reveals modulators of lipid productivity. ACS Chem Biol.

[35] Zhang A, Mernitz K, Wu C, Xiong W, Wang X (2021). ATP drives efficient terpene biosynthesis in marine *Thraustochytrids*. MBio.

[36] Lushchak VI (2011). Adaptive response to oxidative stress: bacteria, fungi, plants and animals. Comp Biochem Physiol C Toxicol Pharmacol.

[37] Wang Z, Cheng J, Zhang X, Chen L, Liu J (2021). Metabolic pathways of *Chlorella* sp. cells induced by exogenous spermidine against nitric oxide damage from coal-fired flue gas. Bioresour Technol.

[38] Yue XH, Chen WC, Wang ZM, Liu PY, Wan X (2019). Lipid distribution pattern and transcriptomic insights revealed the potential mechanism of docosahexaenoic acid traffics in *Schizochytrium* sp. A-2. J Agric Food Chem.

[39] Chokshi K, Pancha I, Ghosh A, Mishra S (2017). Nitrogen starvation-induced cellular crosstalk of ROS-scavenging antioxidants and phytohormone enhanced the biofuel potential of green microalga *Acutodesmus*
*dimorphus*. Biotechnol Biofuels.

[40] Tsang CK, Chen M, Cheng X, Qi Y, Chen Y, Das I, Li X, Vallat B, Fu LW, Qian CN (2018). SOD1 phosphorylation by mTORC1 couples nutrient sensing and redox regulation. Mol Cell.

[41] Li J, Ren LJ, Sun GN, Qu L, Huang H (2013). Comparative metabolomics analysis of docosahexaenoic acid fermentation processes by *Schizochytrium* sp. under different oxygen availability conditions. OMICS.

[42] Siniossoglou S (2013). Phospholipid metabolism and nuclear function: roles of the lipin family of phosphatidic acid phosphatases. Biochim Biophys Acta.

[43] Poonam S, Abhishek G, Sheena K, Ismail R, Faizal B (2016). Combined metals and EDTA control: an integrated and scalable lipid enhancement strategy to alleviate biomass constraints in microalgae under nitrogen limited conditions. Energy Convers Manag.

[44] Ren LJ, Yun F, Li J, Liang Q, He H (2013). Impact of phosphate concentration on docosahexaenoic acid production and related enzyme activities in fermentation of *Schizochytrium* sp. Bioprocess Biosyst Eng.

[45] Qu L, Ji XJ, Ren LJ, Nie ZK, Feng Y, Wu WJ, Ouyang PK, Huang H (2015). Enhancement of docosahexaenoic acid production by *Schizochytrium* sp. using a two-stage oxygen supply control strategy based on oxygen transfer coefficient. Lett Appl Microbiol.

[46] Liu YH, Alimujiang A, Wang X, Luo SW, Li HY (2019). Ethanol induced jasmonate pathway promotes astaxanthin hyperaccumulation in *Haematococcus* pluvialis. Bioresour Technol.

