# Supplementary material for: An emerging simple and effective approach to increase the productivity of thraustochytrids microbial lipids by regulating glycolysis process and triacylglycerols’ decomposition
Source: Biotechnol Biofuels. 2021 Dec 31;14:247. doi: 10.1186/s13068-021-02097-4 (PMC8719115; doi:10.1186/s13068-021-02097-4)
Supplement: Supplementary file 1 — Additional file 1. Additional figures and table. [file 13068_2021_2097_MOESM1_ESM.doc]

**An emerging simple and effective approach to increase the productivity of Thraustochytrids microbial lipids by regulating glycolysis process and** **triacylglycerols decomposition**

Wang Ma^a^, Yu-Zhou Wang^a^, Fang-Tong Nong^a^, Fei Du^a^, Ying-Shuang Xu^a^, Peng-Wei Huang^a^, Xiao-Man Sun*^a^

^a^ School of Food Science and Pharmaceutical Engineering, Nanjing Normal University, 2 Xuelin Road, Qixia District, Nanjing, People’s Republic of China

^b^ College of Biotechnology and Pharmaceutical Engineering, Nanjing Tech University, No. 30 South Puzhu Road, Nanjing, People’s Republic of China

**Correspondence:** [xiaomansun@njnu.edu.cn](mailto:xiaomansun@njnu.edu.cn)


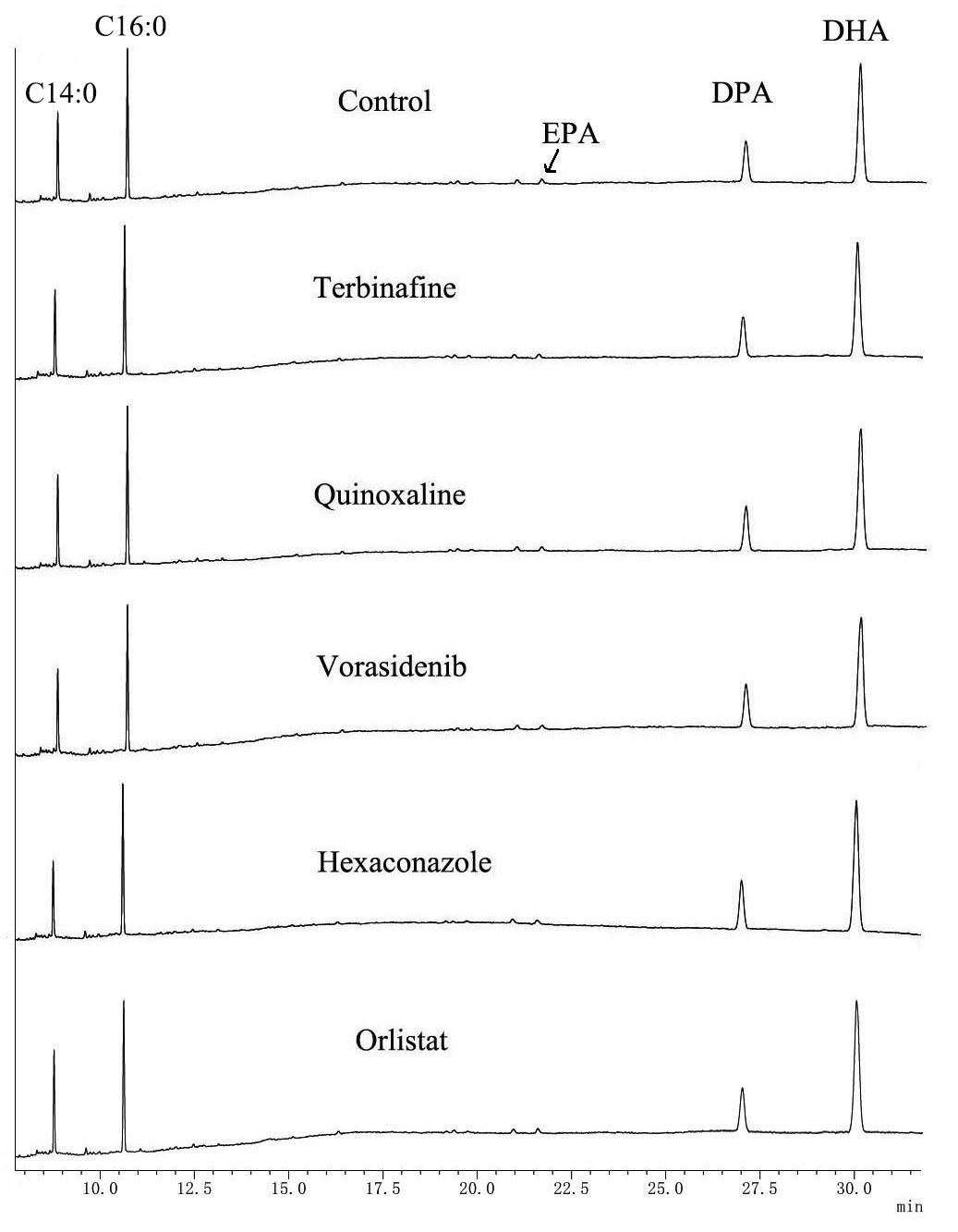


**Fig. S1** Meteorological map of the effects of different chemical modulators on fatty acid composition of *Schizochytrium* sp. HX-308. DPA, Docosapentaenoic acid, C22:5; DHA, Docosahexaenoic acid, C22:6; EPA, eicosapentaenoic acid, C20:5.


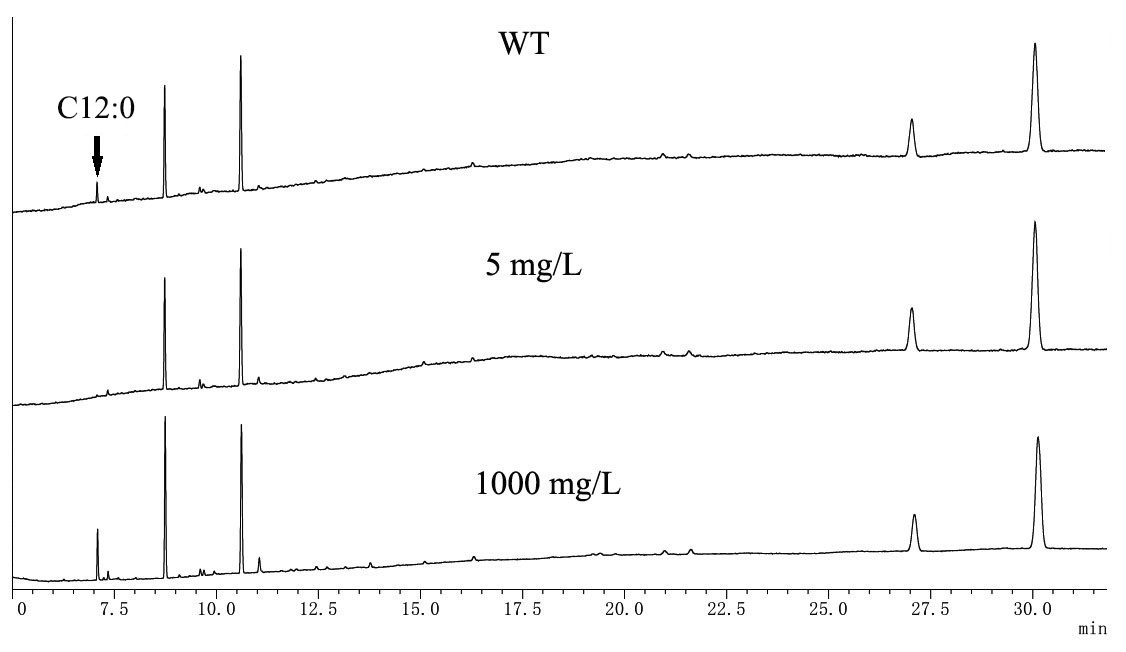


**Fig. S2** Meteorological map of the effects of lipase inhibitor on fatty acid composition of *Schizochytrium* sp. HX-308.


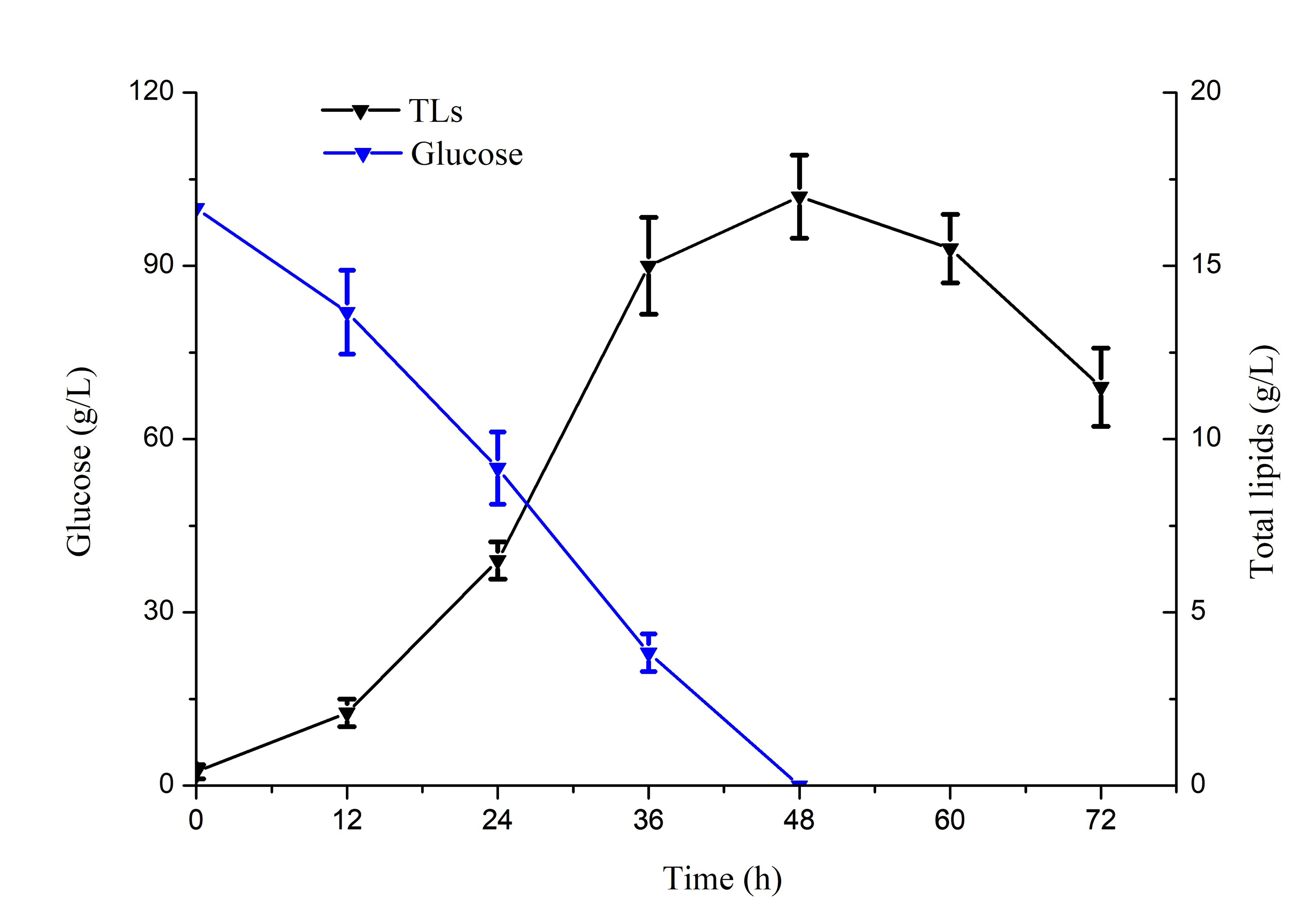


**Fig. S3** Changes in lipid accumulation of *Schizochytrium* sp. HX-308 without fed-feeding fermentation.


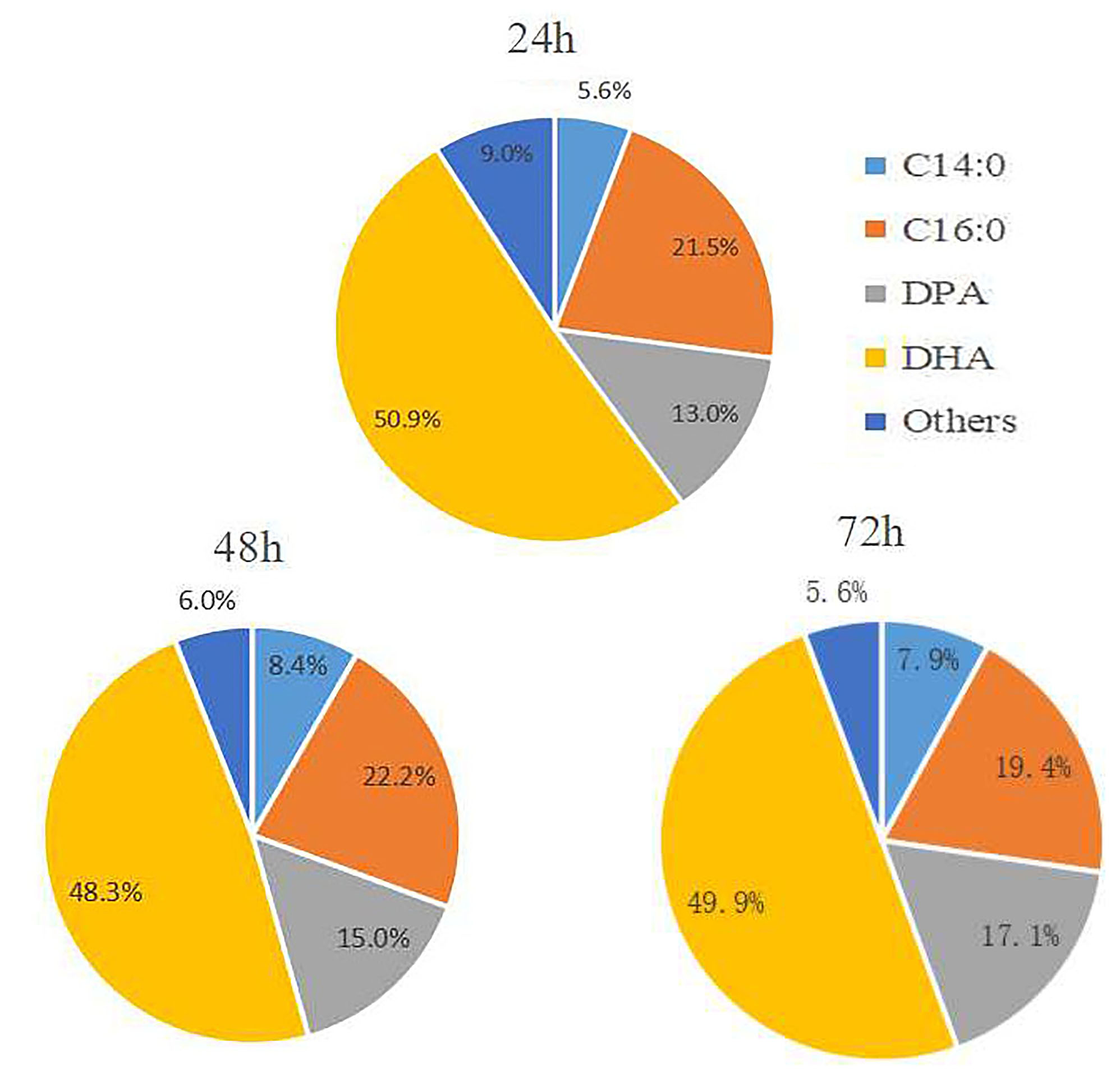


**Fig. S4** Changes in fatty acid composition of *Schizochytrium* sp. HX-308 during different periods of without fed-feeding fermentation.


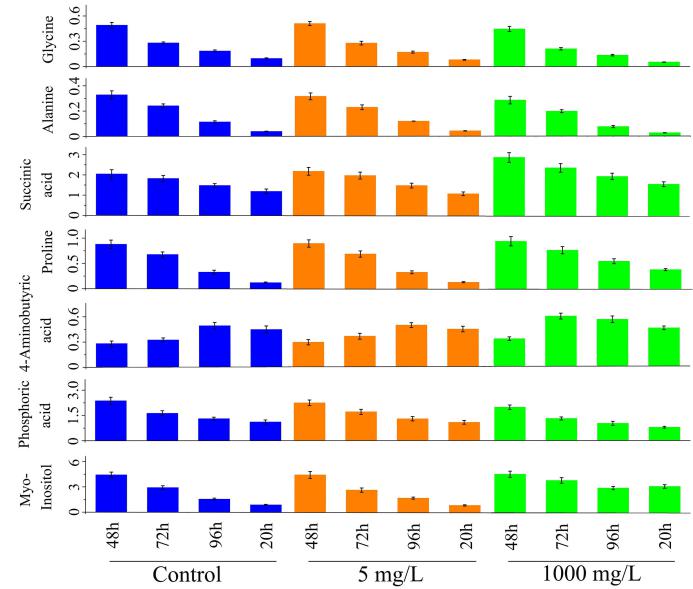


**Fig. S5** Seven representative metabolites changes in *Schizochytrium* sp. HX-308 induced by orlistat under 5 mg/L and 1000 mg/L.


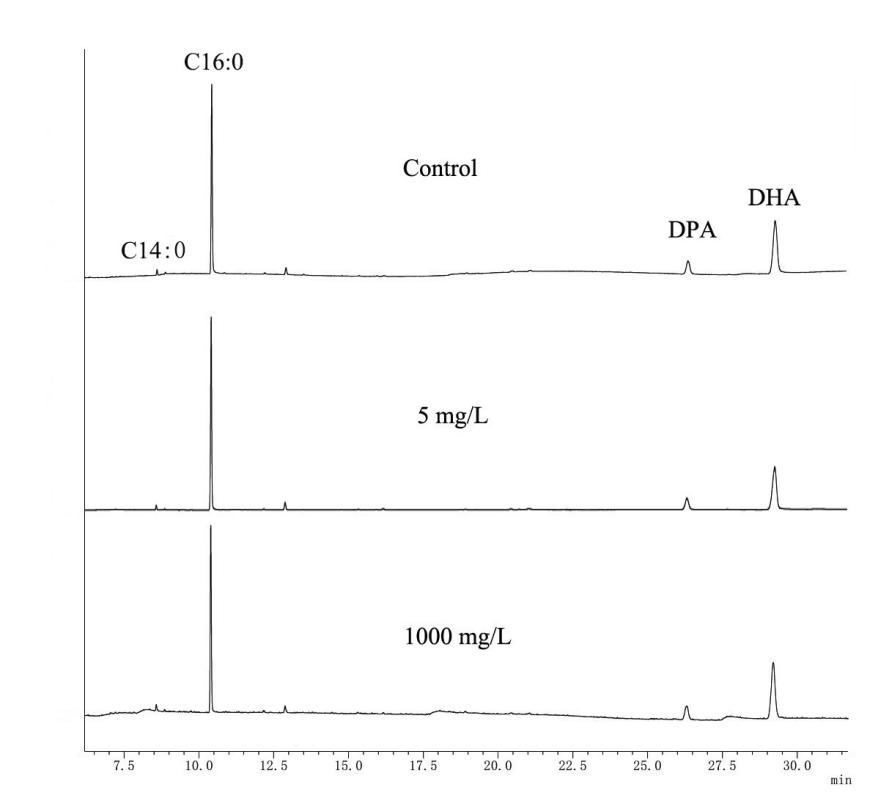


**Fig. S6** Meteorological map of the effects of lipase inhibitor on fatty acid composition of Thraustochytrid *Aurantiochytrium*.


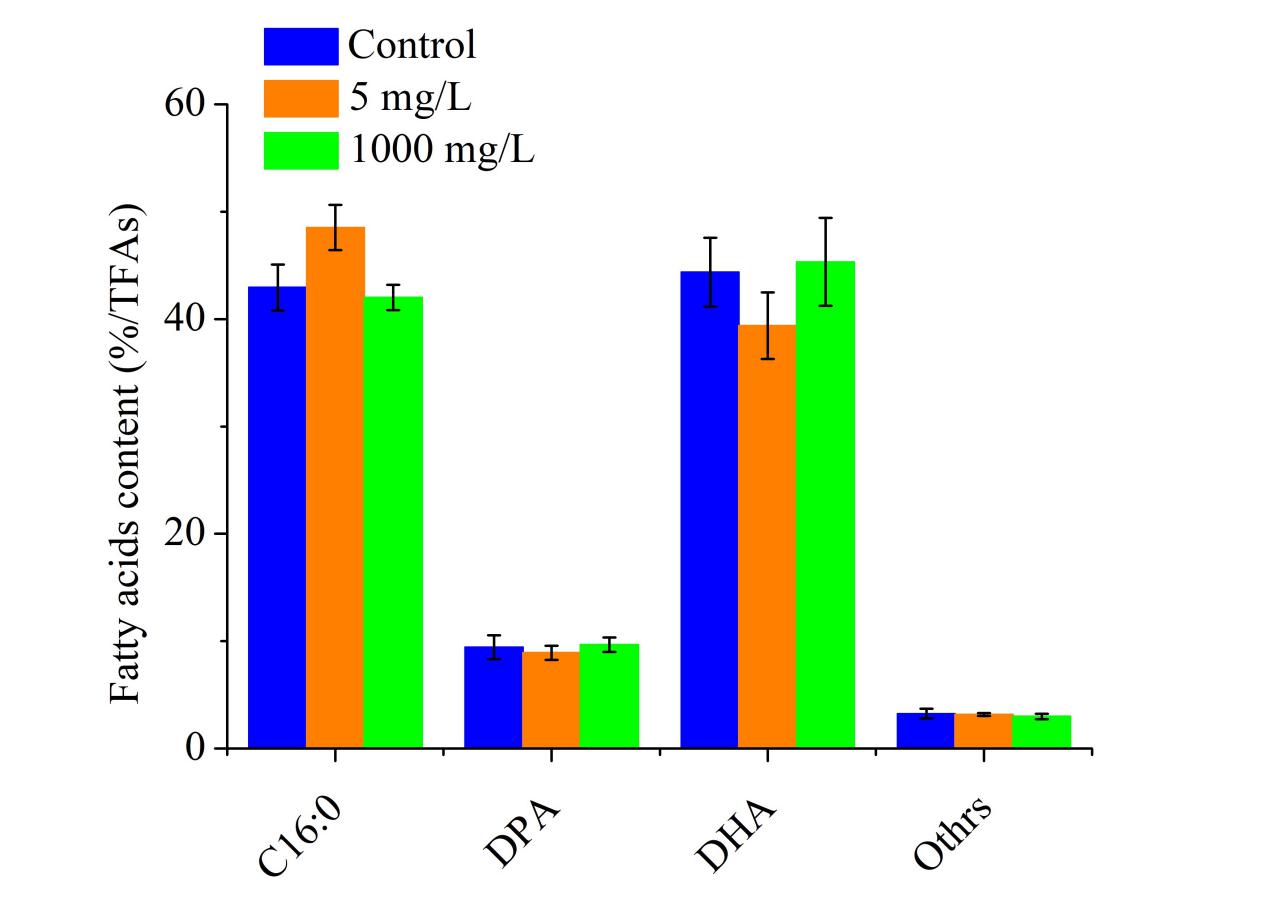


**Fig. S7** The effects of lipase inhibitor on fatty acid content of Thraustochytrid *Aurantiochytrium*.


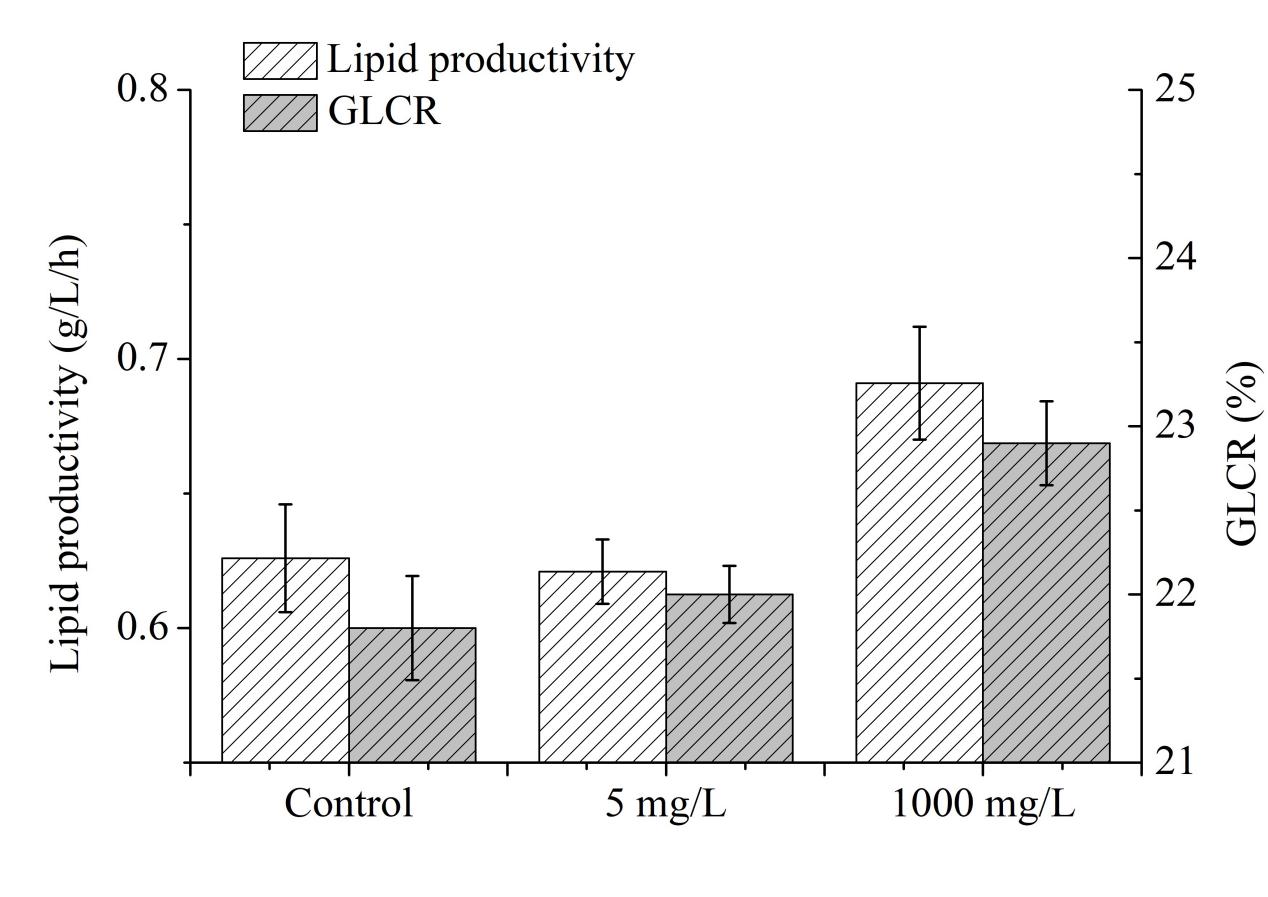


**Fig. S8** The effects of lipase inhibitor on lipid productivity and GLCR of Thraustochytrid *Aurantiochytrium*.


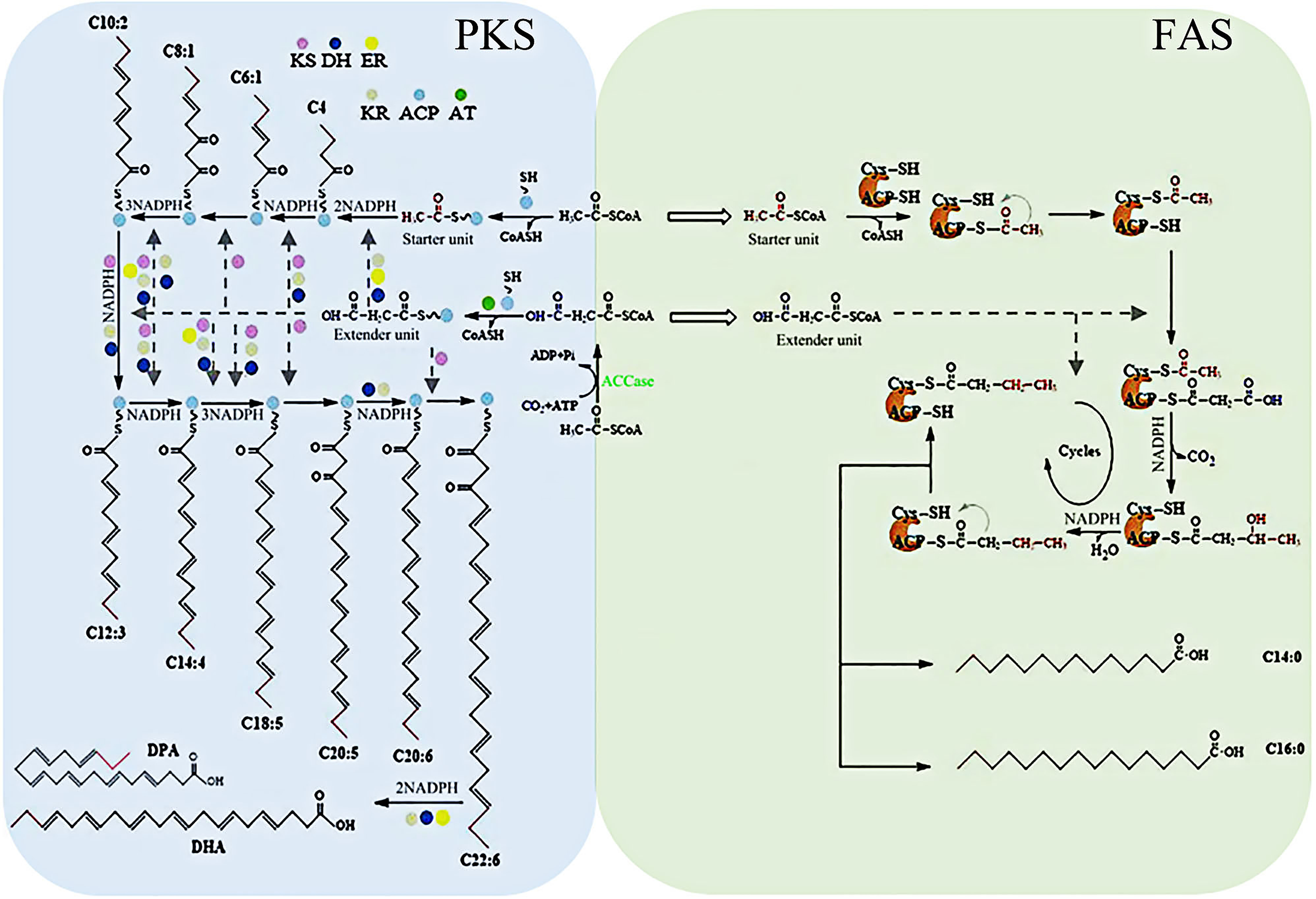


**Fig. S9** The Fatty acid synthesis pathway of *Schizochytrium* sp. HX-308. AT, acyltransferase; ACP, acyl carrier protein; KS, Ketosynthase; DH, dehydratase; ER, enoylreductase; KR, ketoreductase; Cys, Cysteine; FAS, fatty acid synthesis.

**Table S1** Chemical modulators screening strategy.

| **Chemicals** | **Functions** | **Purposes** | **Reference** |
| --- | --- | --- | --- |
| Vorasidenib | ICDH inhibitor | Reduce bypass consumption of acetyl-CoA | Fathi et al. (2018) |
| Quinoxaline | PEPC inhibitor | Increase the supply of acetyl-CoA | Paulus et al. (2014) |
| Hexaconazole | Sterol biosynthesis inhibitor | Reduce bypass consumption of acetyl-CoA | Sun et al. (2019) |
| Terbinafine | SE inhibitor | Reduce bypass consumption of acetyl-CoA | Lu et al. (2014) |
| Orlistat | Glycerolipase inhibitor | Reduce lipid consumption | Franz et al. (2013) |

**References**

Fathi AT, Dinardo CD, Kline I, Kenvin L, Gupta I, Attar EC, Stein EM, Botton SD. Differentiation Syndrome Associated With Enasidenib, a Selective Inhibitor of Mutant Isocitrate Dehydrogenase 2: Analysis of a Phase 1/2 Study. JAMA Oncol. 2018;4:1106-1110.

Paulus JK, Forster K. Groth G. Direct and selective small-molecule inhibition of photosynthetic PEP carboxylase: New approach to combat C4 weeds in arable crops. FEBS letters. 2016;588(12):2101-2106.

Sun XM, Ren LJ, Zhao QY, Zhang LH, Huang H. Application of chemicals for enhancing lipid production in microalgae-a short review. Bioresour. Technol. 2019;293:122135.

Lu Y, Zhou W, Wei L, Li J, Jia J, Li F, Smith SM, Xu J. Regulation of the cholesterol biosynthetic pathway and its integration with fatty acid biosynthesis in the oleaginous microalga Nannochloropsis oceanica. Biotechnol Biofuels. 2014;7(1):1-15.

Franz AK, Danielewicz MA, Wong DM, Anderson LA, Boothe JR. Phenotypic Screening with Oleaginous Microalgae Reveals Modulators of Lipid Productivity. ACS Chem Biol. 2013;8:1053-62.

Q5:We noticed that the transcription level of ME has been improved under the conditions of orlistat, and the activity of ME has a greater impact on PUFAs. So, why is the proportion of PUFAs reduced at 1000 mg/L?

Q6:Why did the transcription levels of ME and G6PDH not decrease, but the NADPH in the cell did decrease?
